# Enhancing Preclinical Rigor: Evaluating Robustness and Numerical Stability in a Chronic Pancreatitis Mouse Model

**DOI:** 10.1111/nyas.70295

**Published:** 2026-06-09

**Authors:** Annika Thämlitz, Muhammad Imran Khan, Benjamin Schulz, Lorenzo Pilch, Brigitte Vollmar, Dietmar Zechner

**Affiliations:** ^1^ Institute for Experimental Surgery University Medical Centre Rostock Rostock Germany

**Keywords:** cerulein‐induced chronic pancreatitis, GSK805 therapy, reliability of research, robustness index, robustness of conclusion, unit fragility index

## Abstract

Chronic pancreatitis (CP) is characterized by progressive fibrosis, inflammation, and pancreatic atrophy. Here, we evaluate a milder CP model using only 42 cerulein injections administered in both male and female C57BL/6J (BL6) and BALB/c mice. We further assess the therapeutic potential of the RORγt inhibitor GSK805. We employed a multi‐tiered analytical approach, including the robustness of conclusion (RC), unit fragility index, and robustness index, to evaluate consistency and numerical stability of conclusions. The model reliably produced key hallmarks of CP: reduced pancreas‐to‐body weight ratio, elevated collagen I deposition, and tubular complex formation with an RC of 100%. In contrast, the induction of *Tnf*, *Ifng*, *Il10*, *Il23r*, *Il6*, and *Il1b* expression was less robust and partially strain specific. *Il23r* and *Il10* were significantly induced during CP in female and male BL6 but not in BALB/c mice. *Il6* was significantly induced in female and male BALB/c but only reached significance in BL6 when sexes were combined. Treatment with GSK805 reduced *Il23r* expression level significantly in BL6 mice, but no reliable improvements in other disease characteristics were observed. Overall, this study validates a simplified, robust CP model and advocates for the integration of complementary statistical metrics to strengthen the reliability of scientific conclusion.

## Graphical abstract

In a modified cerulein‐induced chronic pancreatitis (CP) mouse model, key hallmarks of CP were robustly induced in male and female C57BL/6J (BL6) and BALB/c mice, whereas cytokine responses varied partly according to strain and sex. The RORγt inhibitor GSK805 reduced *Il23r* expression in the BL6 strain, significantly decreased collagen I deposition and tubular complex formation exclusively in male BL6 mice. These findings support the integration of complementary statistical metrics to evaluate the robustness and reliability of scientific conclusions

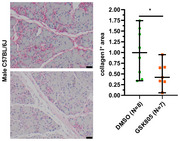

## 1 Introduction

The probabilistic evaluation of data through a single statistical method, typically resulting in a *p* value, has long served as a cornerstone of statistical inference in scientific research. Initially introduced by Fisher and later expanded upon, the *p* value remains a widely adopted metric in hypothesis testing due to its simplicity and interpretability. In contemporary science, *p* values are routinely employed to determine whether observed effects are likely to be due to chance, thereby guiding decisions about the acceptance or rejection of null hypotheses [1]. Nevertheless, the replicability crisis has sparked ongoing debate within the scientific community about severe limitations when solely using *p* values to describe data [2, 3]. For example, the reliance on only one specific statistical method based on an arbitrary threshold for significance can promote practices such as “p‐hacking” and selective reporting, thus undermining scientific integrity [4, 5, 6].

These issues underscore the need for complementary approaches that describe the numeric stability of data sets and the robustness of conclusion (RC). In our study we use the word robustness as defined by Goodman et al. in 2016 [7, 8]. Robustness denotes the stability of conclusions based on distinct data sets when experimental conditions or assumptions are varied [7, 8]. For example, the effect of a drug is considered robust if it produces consistent conclusions despite minor methodological changes. A drug that reduces fibrosis regardless of patient sex, age, or other factors exemplifies robustness. This concept relates to generalizability, which measures how well findings apply across different settings or populations [7].

Complementing robustness, analyzing numerical stability or numerical fragility offers an additional quantitative lens focusing on a data set. For this purpose, Feinstein in 1990 introduced the unit fragility index (UFI) [9]. Feinstein described that data sets can yield identical *p* values; however, their fragility is often different when challenged by misclassifications. This is more likely when the data sets contain small sample sizes, but even clinical studies with large sample sizes have been reported to reach only very fragile conclusions, although a significant *p* value was reported [10, 11]. UFI examines the effect on the *p* value, by transferring one sample within a 2 × 2 contingency table from one to the other group [9, 12]. When many misclassifications are needed to flip the *p* value from significant to nonsignificant, the numerical stability is high and the fragility of the data is low, supporting the hypothesis that conclusions based on this data set might be replicated in future experiments [9, 12].

To address numerical stability/fragility by another method, Heston recently described the robustness index (RI) [13]. Unlike UFI, RI preserves the integrity of the underlying data distribution by adjusting the sample size for all four values in a 2 × 2 contingency table simultaneously by scaling (multiplying or dividing) them with small decimal values, until the *p* value flips. This approach indicates how robust a finding is under proportional transformations [14].

These statistical tools are particularly relevant in preclinical research, where small sample sizes and high variability are common challenges that can undermine the reliability of observed associations. Clinical trials often cannot reproduce preclinical results, so introducing measures for robustness and numeric stability or fragility is essential to improve reliability of preclinical studies. In the context of chronic pancreatitis (CP), a progressive inflammatory disorder characterized by pancreatic atrophy and extensive fibrosis, such methodological rigor is essential [15]. Despite advancements in endoscopic and surgical interventions, progress in effectively treating CP remains modest, with most therapies providing only partial symptom relief and limited reversal of disease progression [16, 17]. The resulting functional decline in CP is closely associated with immunological changes, including elevated expression levels of key inflammatory markers such as cytokine genes *Ifng*, *Tnf*, *Il1b*, *Il6*, and *I10* [18, 19, 20, 21]. To study disease pathology and evaluate novel drugs, CP is often induced in a murine model by administering 6–8 injections of cerulein per day for several weeks [22, 23]. In this murine model, immune modulator genes such as *Il1b*, *Il6*, *Il10*, *Tnf*, and *Ifng* are also induced during CP [18, 24, 25, 26].

The so‐called IL‐23/IL‐17 pathway has been implicated in CP [27]. This pathway is initiated by pro‐inflammatory cytokine IL‐23 and leads to differentiation and expansion of Th17 cells [28, 29]. The transcription factor retinoic acid‐related orphan receptor gamma t (RORγt) is a master regulator of this pathway [30, 31, 32, 33]. RORγt mediates transcription of pro‐inflammatory cytokine and receptor genes [30], including *Il17a*, *Il17f*, *Il22*, and *Il23r*, that contribute to sustained inflammation [31, 34, 35]. The RORγt inhibitor GSK805 has been suggested as a potential therapeutic agent for CP due to its ability to modulate immune responses in autoimmune diseases and chronic cholestasis [36, 37]. This compound inhibits RORγt transcriptional activity. Loss of RORγt function prevents Th17 cell differentiation and expression of proinflammatory cytokines and receptors such as IL‐23R [38, 39].

In our study, we pursued three primary aims. First, we investigated whether CP can be robustly induced using a shortened protocol involving only 42 injections of cerulein. Second, we assessed the therapeutic potential of RORγt inhibitor GSK805 by quantitatively assessing fibrosis, histopathological changes, and cytokine expression profiles. Third, we evaluated the robustness and numerical stability of our findings. To address RC, we analyzed both male and female mice across two genetically distinct strains, BL6 and BALB/c, creating four independent experimental conditions to capture sex‐ and strain‐related variability. In addition, we rigorously assessed numerical stability using two complementary metrics, UFI and RI. Together, these analyses demonstrate how integrating robustness and numeric stability metrics enhances the interpretation of complex biological data, providing a more reliable foundation for drawing conclusions in preclinical research.

## 2 Materials and Methods

### 2.1 Mouse Strains and Animal Husbandry

BALB/cAnNCrl (BALB/c) and C57BL/6J (BL6) mice were bred under specified pathogen‐free conditions in our animal facility. During routine health monitoring of the animal stock, *Helicobacter* spp., *Rodentibacter heylii*, and murine norovirus were detected in a small number of animals, which were excluded from this study. Throughout the experiment, all mice were single‐housed in Type III cages (Zoonlab GmbH, Castrop‐Rauxel, Germany) under standardized conditions: a 12‐h light–dark cycle, a temperature of 21°C ± 2°C, and relative humidity of 60% ± 20%. The mice were provided with tap water and food pellets (10 mm, ssniff‐Spezialdiäten GmbH, Soest, Germany) ad libitum. Environmental enrichment included a paper roll (75 × 38 mm^2^, H 0528‐151, ssniff‐Spezialdiäten GmbH), nesting material (shredded tissue paper, Verbandmittel GmbH, Frankenberg, Germany), and a wooden stick (40 × 16 × 10 mm^3^, Abedd, Vienna, Austria).

### 2.2 Animal Experiments

CP was induced in mice through repetitive intraperitoneal (i.p.) injections of cerulein (Bachem, Bubendorf, Switzerland; product no. 4030451) dissolved in 0.9% sodium chloride. The injections (50 µg/kg) were administered three times per day, at hourly intervals, on days 0, 2, 4, 7, 9, 11, 14, 16, 18, 21, 23, 25, 28, and 30. This schedule resulted in 42 total injections (i.e., 14 × 3/day), over a span of 30 days.

To evaluate the potential therapeutic effects of an RORγt inhibitor, half of the mice were treated with GSK805 (Hycultec, Beutelsbach, Germany; product no. Hy‐12776). GSK805 was prepared by dissolving it at a concentration of 40 mg/mL in 100% DMSO, followed by daily preparation of a 10‐fold dilution in corn oil. The diluted solution was administered via oral gavage at a dose of 30 mg/kg daily, from days 1 to 31 following the initial cerulein injection. Control mice received 100% DMSO diluted 10‐fold in corn oil under the same administration protocol. Animal treatments were conducted following randomization and were performed in a blinded manner to ensure unbiased assessment.

A total of 84 mice were used in this study. As healthy control mice, five female and five male C57BL/6J mice and five female and five male BALB/c mice, aged 15–24 weeks, were used (=20 mice). CP was induced in 64 mice. Three mice were euthanized before the end of the experiment owing to humane considerations: one GSK805‐treated male BL6 mouse, one DMSO‐treated female BALB/c mouse, and one GSK805‐treated female BL6 mouse. Data from these animals were excluded from analysis. Decisions to euthanize were based on a previously published distress scoring system [40]. Criteria for euthanasia included signs such as persistent cramping, abnormal respiratory sounds, marked apathy, or a body weight loss of more than 20%. Additionally, mice were euthanized if their combined distress score exceeded 15 points (out of a maximum possible score of 66 points). Data from 61 CP mice were included in the data analysis: 15 female BL6 mice (16–21 weeks old; 7 treated with GSK805 and 8 treated with DMSO as vehicle control), 15 male BL6 mice (16–21 weeks old; 7 treated with GSK805 and 8 treated with vehicle), 15 female BALB/c mice (15–22 weeks old; 8 treated with GSK805 and 7 treated with vehicle), and 16 male BALB/c mice (15–21 weeks old; 8 treated with GSK805 and 8 treated with vehicle).

All mice received analgesia by adding 3 g/L metamizole (Ratiopharm GmbH, Ulm, Germany) to their drinking water, which was refreshed daily. Analgesic treatment began 1 week prior to the first cerulein injection (day 0) and continued until day 32, when tissue collection was performed. All animal experiments were approved by the German local authority, the Landesamt für Landwirtschaft, Lebensmittelsicherheit und Fischerei Mecklenburg‐Vorpommern (approval numbers: AZ 2‐022/19 and 1‐070/20) and were conducted in compliance with ethical guidelines and national law.

### 2.3 qPCR and Immunohistochemistry

For quantitative real‐time polymerase chain reaction (TaqMan RT‐qPCR), half of the pancreas head was rapidly frozen in liquid nitrogen and stored at −80°C. RNA was extracted from the pancreas of each mouse using QIAzol lysis reagent and the RNeasy Mini Kit (both from Qiagen, Hilden, Germany). Complementary DNA (cDNA) was synthesized using the High‐Capacity cDNA Reverse Transcription Kit (Applied Biosystems, Waltham, MA, USA).

The calibrator consisted of an RNA pool isolated from all DMSO‐treated male C57BL/6J mice. After cDNA synthesis, TaqMan RT‐qPCR was performed. TaqMan Gene Expression Assays from Thermo Fisher Scientific (Waltham, MA, USA) were used to quantify the expression of the following genes: *Tnf* (Mm00443258_m1), *Ifng* (Mm99999071_m1), *Il10* (Mm00439614_m1), *Il23r* (Mm00519943_m1), *Il6* (Mm99999064_m1), and *Il1b* (Mm00434228_m1), with *Gapdh* (Mm99999915_g1) serving as the reference gene.

Ct values were calculated using QuantStudio software (Applied Biosystems, Waltham, MA, USA). The ∆Ct values were determined as the difference between the Ct value of the gene of interest and the Ct of the reference gene (Ct_gene of interest_ − Ct_reference gene_). Subsequently, ∆∆Ct values were calculated as the difference between ∆Ct of the sample and the ∆Ct of the calibrator (∆∆Ct = ∆Ct − ∆Ct_calibrator_). Fold changes in gene expression levels were calculated using the 2^(−∆∆Ct) method.

For histopathological evaluations, the pancreas tail was rapidly removed and fixed in formalin (Grimm Med. Logistik GmbH, Torgelow, Germany). The tissue was then dehydrated, embedded in paraffin, and sectioned. Pancreatic tissue sections were stained with hematoxylin and eosin or subjected to immunohistochemical analysis using a rabbit anti‐collagen I antibody (Abcam, Cambridge, United Kingdom; Cat# ab270993, RRID: AB_2927551) at a 500‐fold dilution. Primary antibody detection was achieved using a phosphatase‐conjugated goat anti‐rabbit secondary antibody (Abcam, Cat# ab97048, RRID: AB_10680574), applied at 200‐fold dilution. For all quantitative assessments, slides were blinded. For collagen I evaluation, 15 photos (12 at the edge of the tissue and 3 from the middle of the tissue) were taken at 200× magnification using a BX51 microscope (Olympus, Hamburg, Germany) and an SC50 camera (Olympus). For the evaluation of tubular complexes, entire tissue sections were scanned in using an Axioscope 7 (Zeiss, Oberkochen, Germany) and an Axiocam 208 color camera (Zeiss). Using QuPath 5.0.0 [41], the total tissue area and the areas of tubular complexes or collagen^+^ regions were quantified. The percentage of the collagen^+^ or tubular complex^+^ area relative to the total tissue area was calculated.

### 2.4 Statistics

Excel 2016 (Microsoft, Redmont, USA) was used for data assembly and calculations. Fold changes in gene expression levels, pancreas‐to‐body weight (PW/BW) ratio, and collagen I deposition were normalized to the mean fold change of the respective control mice with identical sex and genetic background (i.e., healthy animals for CP analyses and DMSO‐treated animals for GSK analyses, respectively). Normalization was performed using the formula: normalized data = data_exp_/mean_control_. GraphPad Prism 8.4.3 (GraphPad Software, San Diego, USA) and R Studio [42] were used to perform the statistical analysis. The Shapiro–Wilk test was applied to assess normality. When the data followed a normal distribution, Student's *t*‐test was used to determine the *p* values. If the data did not meet the normality assumption, the Mann–Whitney *U* test was applied. A *p* value of <0.05 was considered statistically significant. To quantify Cohen's *d* the effect size package in R (Cohens_*d*) was used. In CP analyses, the factor coding was set so that a positive *d* means the CP group had higher values than the healthy group. For GSK analyses, a positive *d* value reflects higher values in mice treated with GSK805 compared to those given DMSO controls. As a general reference, values of *d* <0.2, <0.5, and >0.8 were regarded as representing small, medium, and large effects, respectively.

RC was evaluated across four independent groups (BL6 male, BL6 female, BALB/c male, and BALB/c female). A conclusion was deemed robust if it was consistent across all four groups, with robustness expressed as the percentage of concordant results (e.g., 100% = 4/4).

To access the numeric stability of the study findings, the data were categorized into 2 × 2 contingency tables. The number of healthy mice and those with CP were categorized based on whether they fall below or above the median values observed in healthy mice. Similarly, the number of CP mice treated with DMSO versus those treated with GSK805 were categorized based on whether they fell below or above the median values observed in the DMSO‐treated group.

UFI was then calculated as described by Feinstein using the *fisher.test* function from the *stats* package in R. UFI is derived by transferring a single sample from one group to another (+1 and −1 adjustments), with simultaneous adjustments to maintain the marginal totals. The original *p* value determined by Fisher's exact test defines which cells to modify. If the initial *p* value is ≤0.05 (indicating significance), the smallest observed outcome is incrementally increased by one unit at each iteration until the *p* value becomes nonsignificant (e.g., see Table S1). If the original *p* value is >0.05, the cell with the largest value is increased incrementally by one unit until significance (*p* ≤ 0.05) is achieved (e.g., see Table S2). Thus, UFI is defined as the number of iterations required to change the *p* value from significant to nonsignificant, or vice versa. It has been suggested that a UFI value of >2 indicates numeric stability for a research study [12].

The RI was calculated as described by Heston [13] using the *chisq.test* function from the R *stats* package in R. The original and subsequent *p* values were calculated using the Chi‐square test, as it can handle decimal values [14]. Before performing the Chi‐square test the Haldane correction, which involves adding 0.5 to all cells of the contingency table, if any of the cell values is zero, was used [43]. If the initial *p* value was ≤0.05, each value of the matrix was divided by a decimal value until the *p* value was >0.05 (e.g., see Table S3). Thus, in this case, RI is the smallest divisor that could flip the *p* value from ≤0.05 to >0.05. If initial *p* value was >0.05, each matrix value was multiplied by a decimal value until the *p* value was <0.05 (e.g., see Table S4). Thus, in this case the RI is the smallest multiplier that could flip the *p* value from >0.05 to ≤0.05. When evaluating the smallest multiplier or divisor, we tested decimal values above 1 that differed by an increment of 0.0000000001. Lower increments did not have greater precision, because they provided identical RI values. It has been suggested that an RI >2 indicates a robust finding [13].

## 3 Results

**FIGURE 1:**
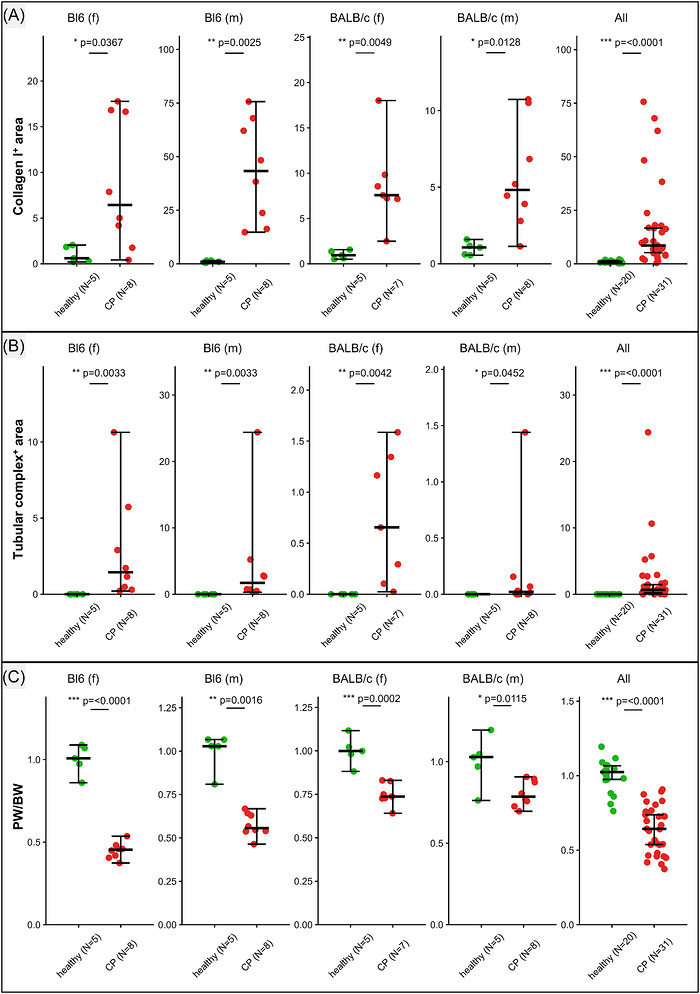
Assessment of key histological and morphometric parameters. Collagen I positive area (A), tubular complex positive area (B), and pancreas weight‐to‐body weight (PW/BW) ratio (C) were quantified in the pancreas of healthy and DMSO‐treated mice with chronic pancreatitis (CP) across mouse strains and sexes. Tubular complex data are presented as % of TBC^+^ area, whereas all other data were normalized to the mean of healthy mice with identical sex and genetic background. Statistical significance is reported as exact *p* values above brackets and denoted by *p < 0.05, **p < 0.01, ***p < 0.001, and ns (not significant). BL6 = C57BL/6J, f = female, m = male.

**FIGURE 2:**
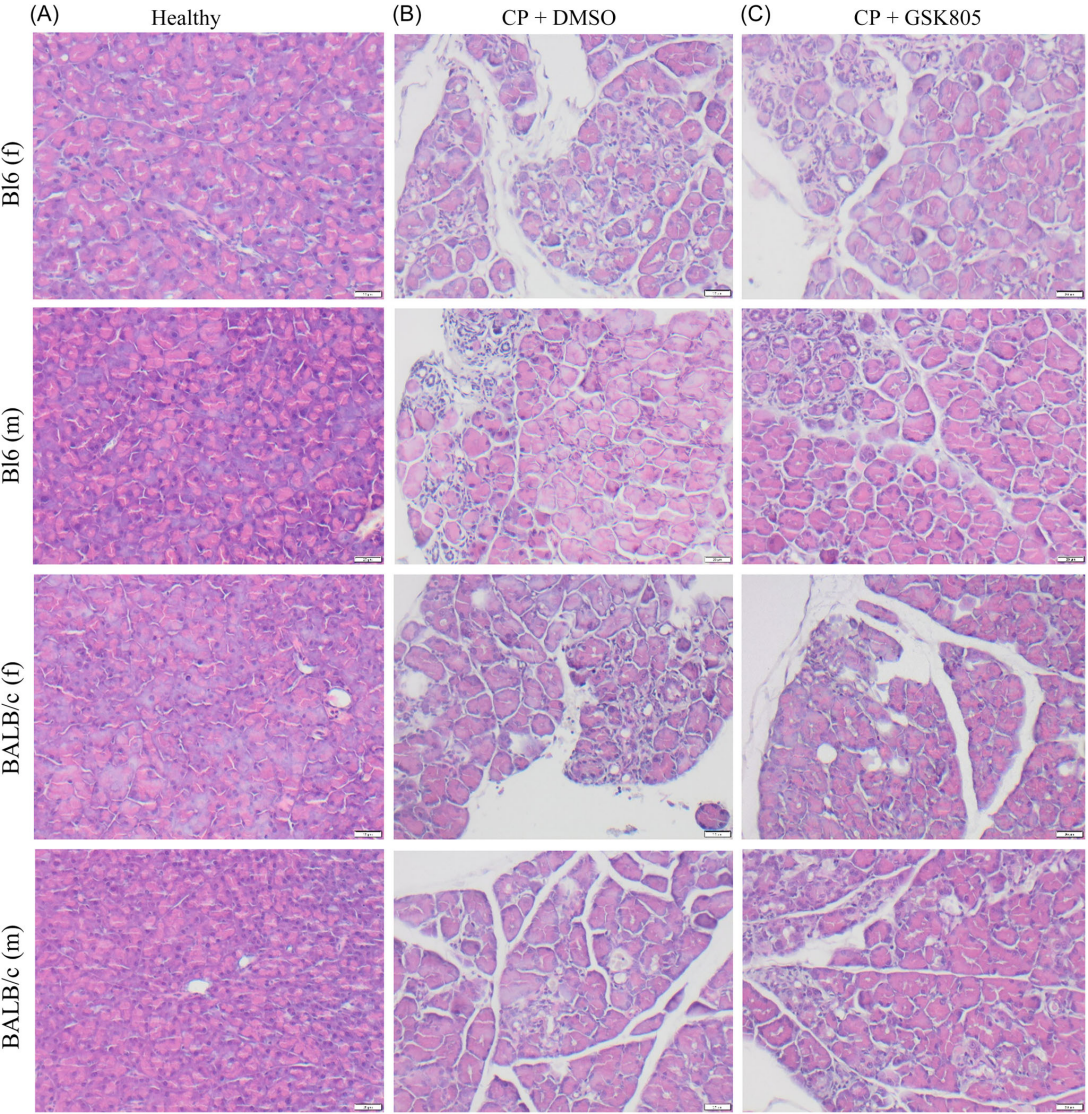
H&E‐stained pancreatic sections. Representative pancreatic sections stained with eosin for cytoplasm (pink), and counterstained with hematoxylin (blue), indicating pseudo tubular complex formation. Columns show tissue from healthy controls (A), mice with cerulein‐induced chronic pancreatitis (CP) treated with DMSO as vehicle control (B), and mice with CP treated with GSK805 (C). BL6 = C57BL/6J, f = female, m = male. Scale bar: 20 µm.

**FIGURE 3:**
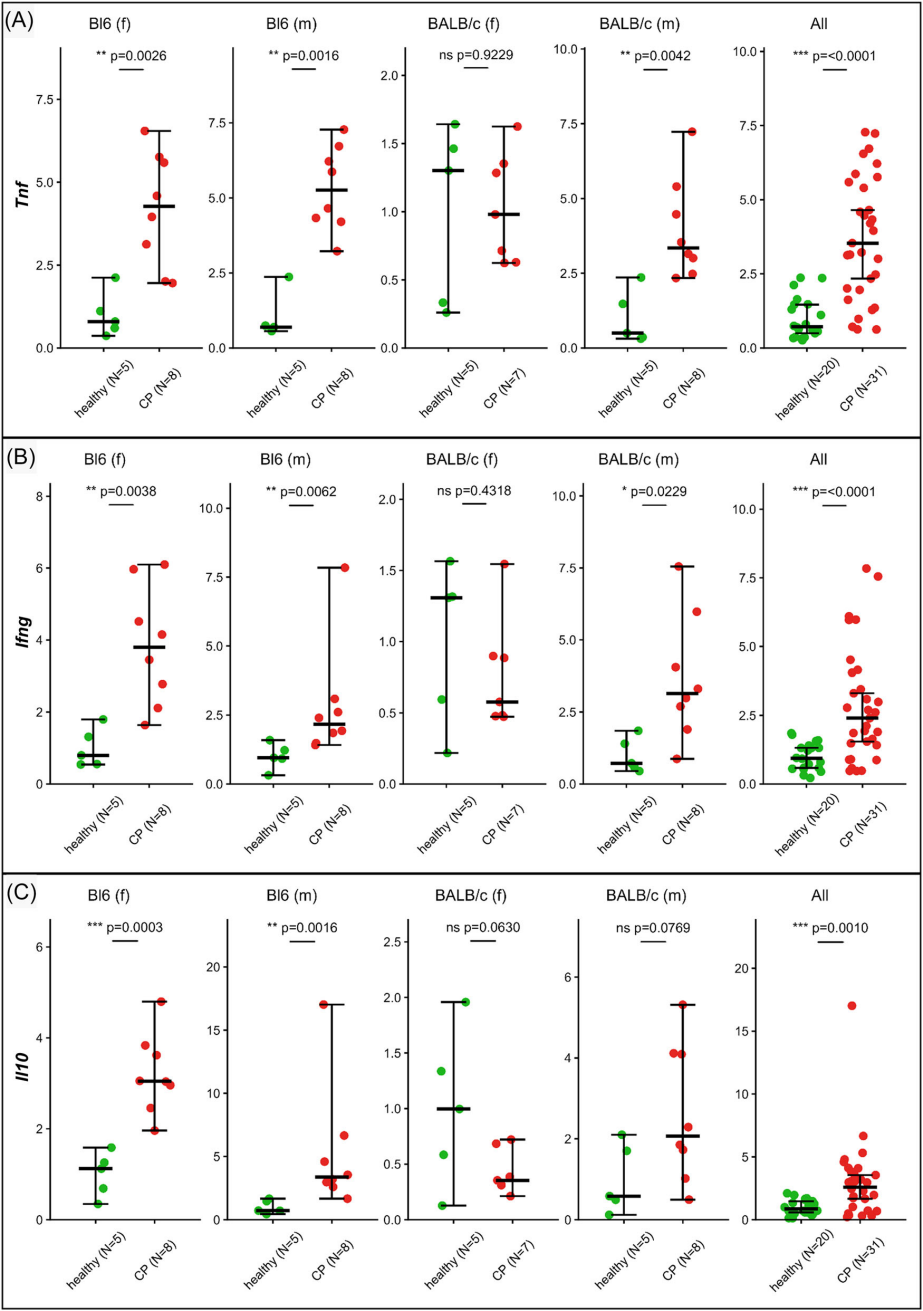
Increase of cytokine expression levels. *Tnf* (A), *Ifng* (B), and *Il10* (C) in the pancreas of healthy and DMSO‐treated mice with chronic pancreatitis (CP) across mouse strains and sexes. All data are presented as fold of mean from healthy mice. Exact *p* values are displayed above brackets; significance levels are denoted by *p < 0.05, **p < 0.01, ***p < 0.001, and ns (not significant). BL6 = C57BL/6J, f = female, m = male.

**FIGURE 4:**
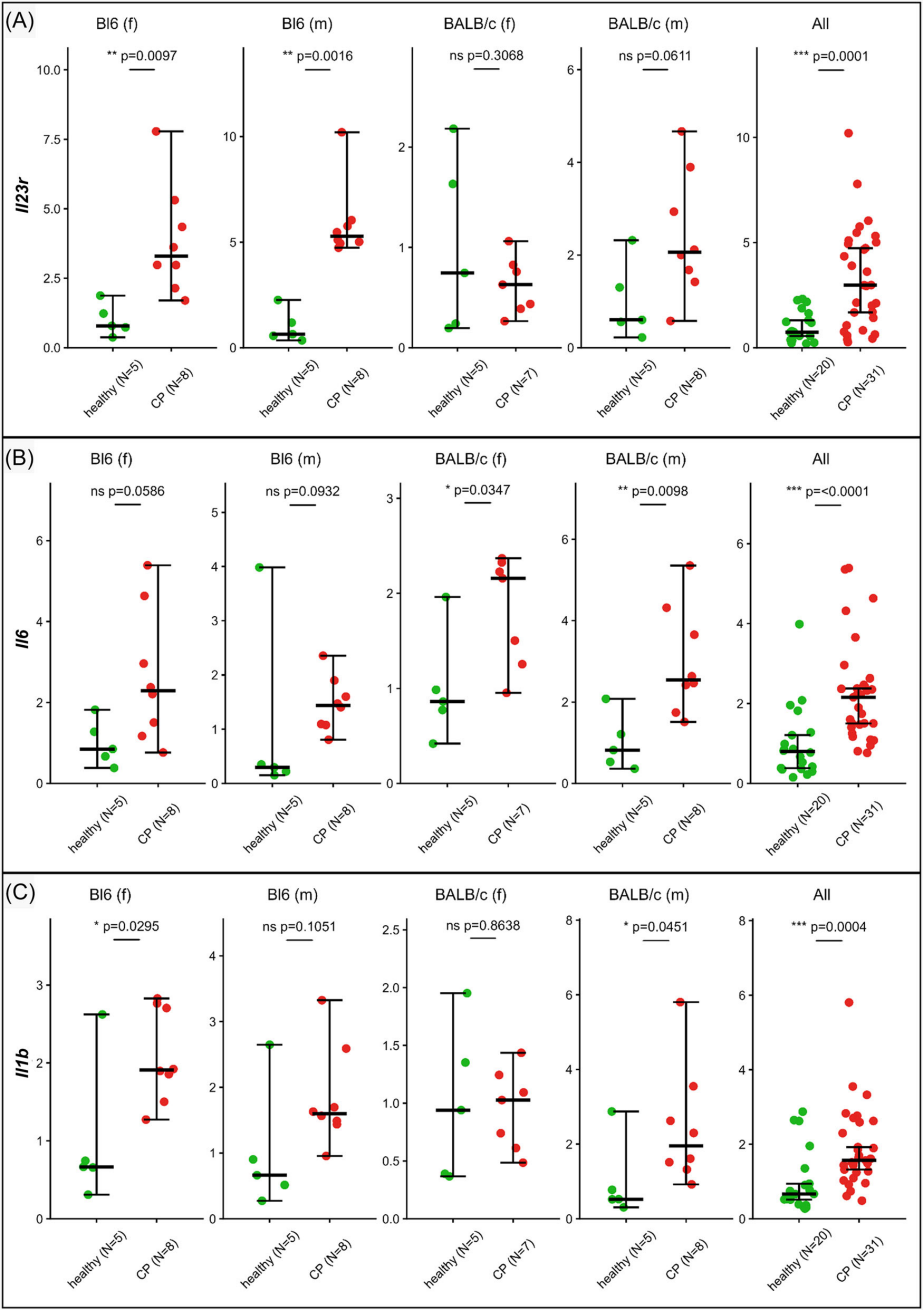
Induction of an interleukin receptor and interleukins. Evaluation of *Il23r* (A), *Il6* (B), and *Il1b* expression (C) in the pancreas of healthy and DMSO‐treated mice with chronic pancreatitis (CP) across mouse strains and sexes. All data are presented as fold of mean from healthy mice. Exact *p* values are shown above brackets; significance is denoted by *p < 0.05, **p < 0.01, ***p < 0.001, and ns (not significant). BL6 = C57BL/6J, f = female, m = male.

The main characteristics of CP, including tissue remodeling, pancreatic atrophy, and inflammation, were compared between cerulein‐treated mice on day 32 of disease progression and healthy control mice (Figures 1, 2, 3, 4, Figures S1 and S2, Table S5).

Fibrosis was assessed by quantifying collagen I deposition in pancreatic tissue (representative sections are shown in Figure S1). Cerulein‐treated animals had significantly more collagen I compared to healthy control animals (Figure 1A, Table S5). Importantly, all four cohorts (female BL6, male BL6, female BALB/c, and male BALB/c) consistently demonstrated significantly higher levels of collagen I in cerulein‐treated mice demonstrating an RC of 100% (Table S5). When data of both sexes were combined, collagen I deposition was also significantly elevated in both mouse strains (Figure S2A). Furthermore, all comparisons showed large effect sizes, with Cohen's *d* values exceeding 1.00, indicating substantial biological differences between groups (Table S5).

Hematoxylin and eosin‐stained sections of pancreas demonstrate preserved acinar architecture with no tubular complex formation in healthy mice. In contrast, CP mice of both strains and sexes display significant tissue remodeling, characterized by pronounced tubular complexes (representative sections are shown in Figure 2). Quantitative analysis of percentage of tubular complex–positive area indicates a statistically significant increase in CP mice compared to healthy controls across all subgroups (Figure 1B and Figure S2B, Table S5). The induction of tubular complex formation was characterized by medium‐large effect sizes and an RC of 100% (Table S5).

The PW/BW ratio, which indicates pancreatic atrophy, was significantly lower in cerulein‐treated mice compared to healthy controls, irrespective of sex or mouse strain (Figure 1C and Figure S2C, Table S5). Notably, BL6 mice exhibited a greater reduction in this ratio, with females showing a decrease to 45.4% (Cohen's *d* = −8.17) and males to 55.6% (Cohen's *d* = −5.02) of the control values. In contrast, BALB/c mice displayed a more moderate reduction, with females at 73.7% (Cohen's *d* = −3.44) and males at 78.5% (Cohen's *d* = −1.72) of the control values (Table S5). When combining all animal cohorts, a highly significant reduction in PW/BW ratio was observed. Again, this was observed independent from sex and mouse strains with a robustness of 100% (Table S5).

For assessing inflammation, the expression of various cytokines and the interleukin receptor 23 gene (*Il23r*) were quantified. When analyzing *Tnf* and *Ifng* expression in the pancreas, significantly more expression was detected in female and male BL6 as well as male BALB/c mice after cerulein injections (Figure 3A,B and Table S5). No significant increase of expression was observed in female BALB/c mice. Thus, repeated cerulein injection leading to significantly increased *Tnf* or *Ifng* expression in the pancreas reached a robustness of 75% (Table S5). When combining data from both sexes, the strain‐specific analysis indicated that BL6 had a notably higher activation of *Tnf* and *Ifng* (Figure S2D,E).

When analyzing *Il10* or *Il23r* expression in the pancreas, significantly more expression was detected in female and male BL6 mice, compared to female and male BALB/c mice, during CP (Figures 3C and 4A, Table S5). Thus, repeated cerulein injection was associated with significantly increased expression of these genes, with a robustness of 50% and mainly observed in BL6 mice (Figure S2F,G and Table S5). When combining data from all animal cohorts, a significant increase in expression of *Il10* and *Il23r* was observed (Figures 3C and 4A).

*Il6* expression in the pancreas was significantly higher in female and male BALB/c mice, compared to female or male BL6 mice, during CP (Figure 4B, Table S5). Thus, repeated cerulein injection lead to significantly increased *Il6* expression, with a robustness of 50%. When combining data from both sexes (Figure S2H) and all of the animal cohorts (Figure 4B), significantly increased expression of *Il6* was observed during CP.

When analyzing *Il1b* expression in the pancreas, significantly more expression was detected in female BL6 and male BALB/c during CP, compared with male BL6 and female BALB/c mice (Table S5, Figure 4C). Thus, repeated cerulein injection was associated with significantly increased *Il1b* expression in the pancreas, with a robustness of 50%. When data from all animal cohorts were combined, a significant increase in expression levels was noticed (Figure 4C). However, the effect was significant only in the BL6 strain (Figure S2I).

**FIGURE 5:**
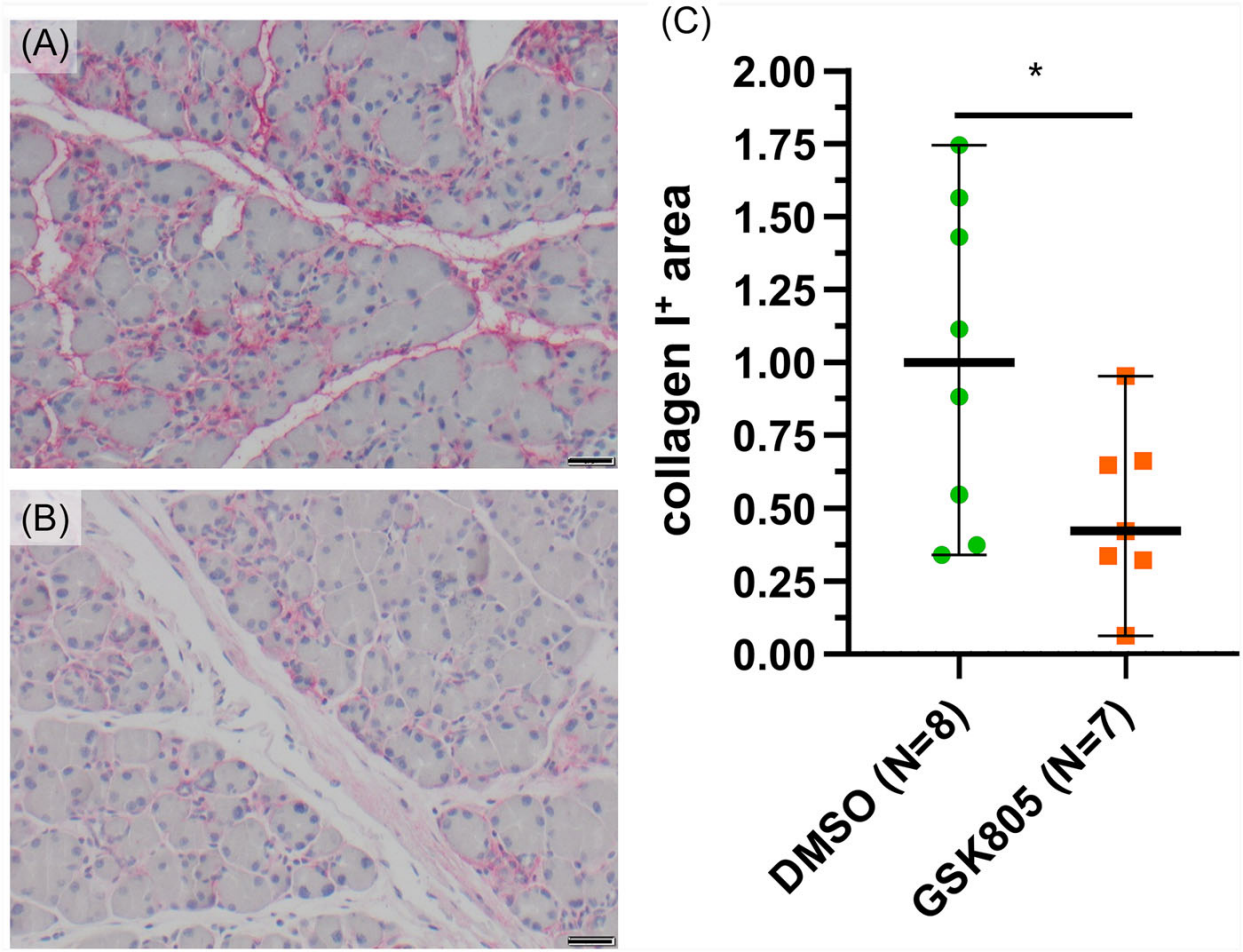
Cerulein‐induced collagen deposition in the pancreas of male C57BL/6 mice. Representative histological sections stained for collagen I (red) from male C57BL/6 mice with cerulein‐induced chronic pancreatitis either treated with vehicle (DMSO) solution (A) or treated with GSK805 (B). Scale bar: 20 µm. Quantitative analysis: percentage of collagen I^+^ area normalized to the mean of DMSO treated mice (C) using an unpaired *t*‐test (*p < 0.05). The graph presents median and 95% confidence interval.

**FIGURE 6:**
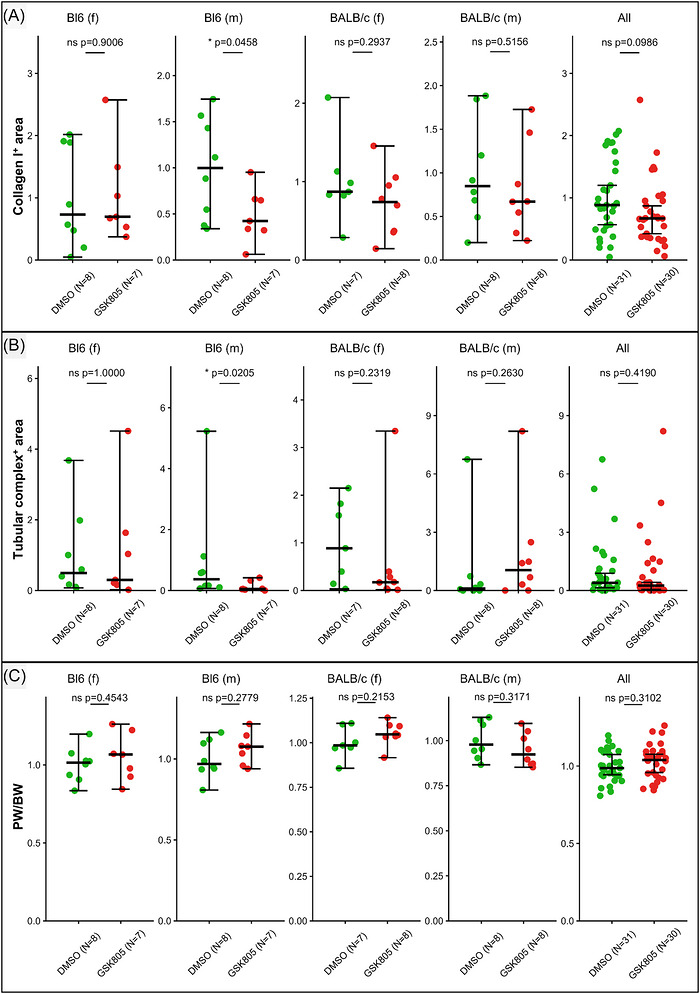
Effect of GSK805 on histological and morphometric parameters. Scatter plots comparing mice with chronic pancreatitis treated with DMSO or GSK805 for collagen I positive area (A), tubular complex positive area (B), and pancreas weight‐to‐body weight (PW/BW) ratio (C), stratified by strain and sex. Tubular complex data are presented as % of TBC^+^ area, whereas all other data were normalized to the mean of DMSO treated mice with identical sex and genetic background. Exact *p* values are shown above brackets; significance is denoted by *p < 0.05 and ns (not significant). BL6 = C57BL/6J, f = female, m = male.

**FIGURE 7:**
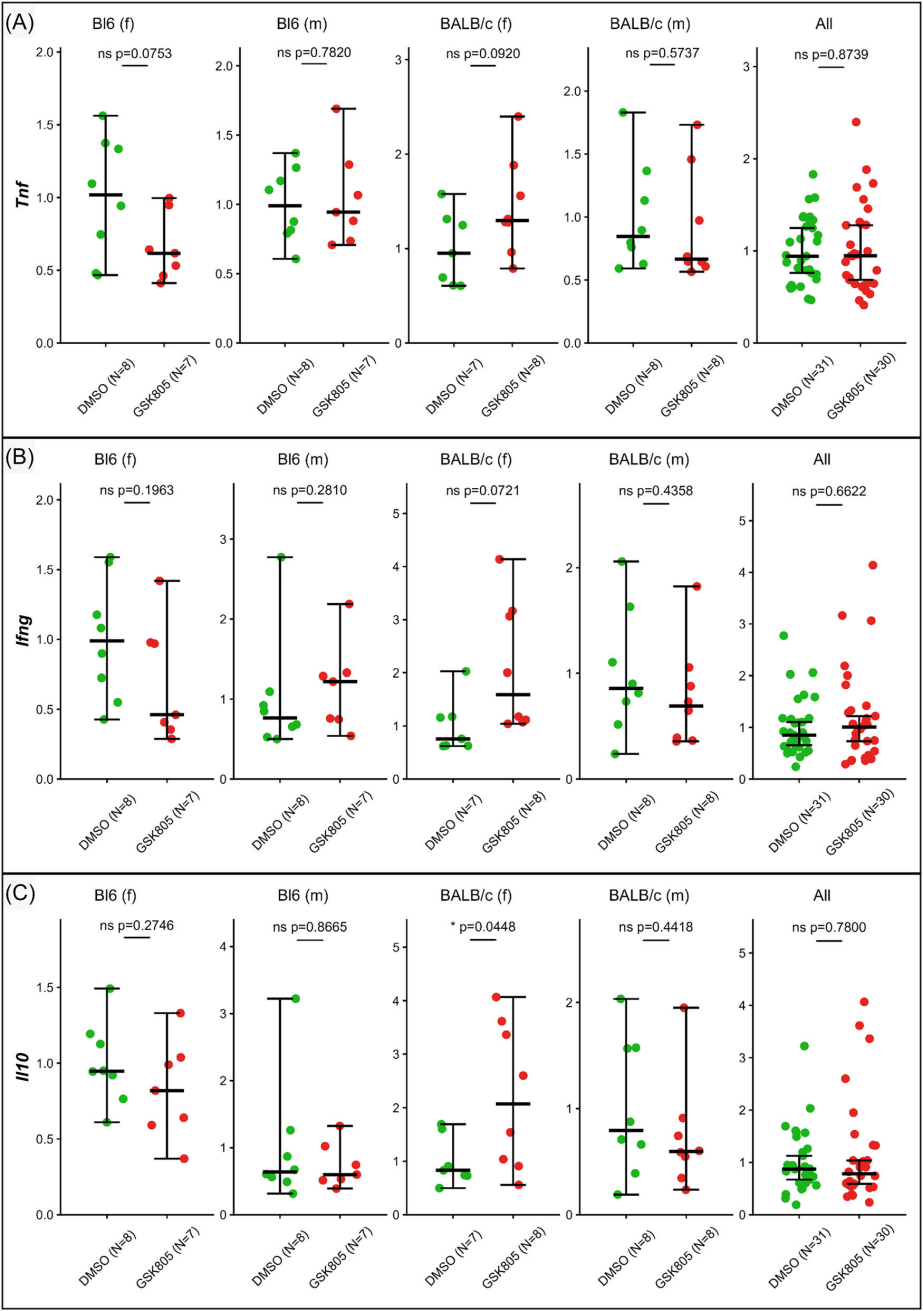
Effects of GSK805 treatment on cytokine expression levels. Evaluation of *Tnf* (A), *Ifng* (B), and *Il10* (C) expression across strains and sexes in mice with chronic pancreatitis treated with DMSO or GSK805. All data are presented as fold of mean from DMSO treated mice. Exact *p* values are shown above brackets; significance is denoted by *p < 0.05 and ns (not significant). BL6 = C57BL/6J, f = female, m = male.

**FIGURE 8:**
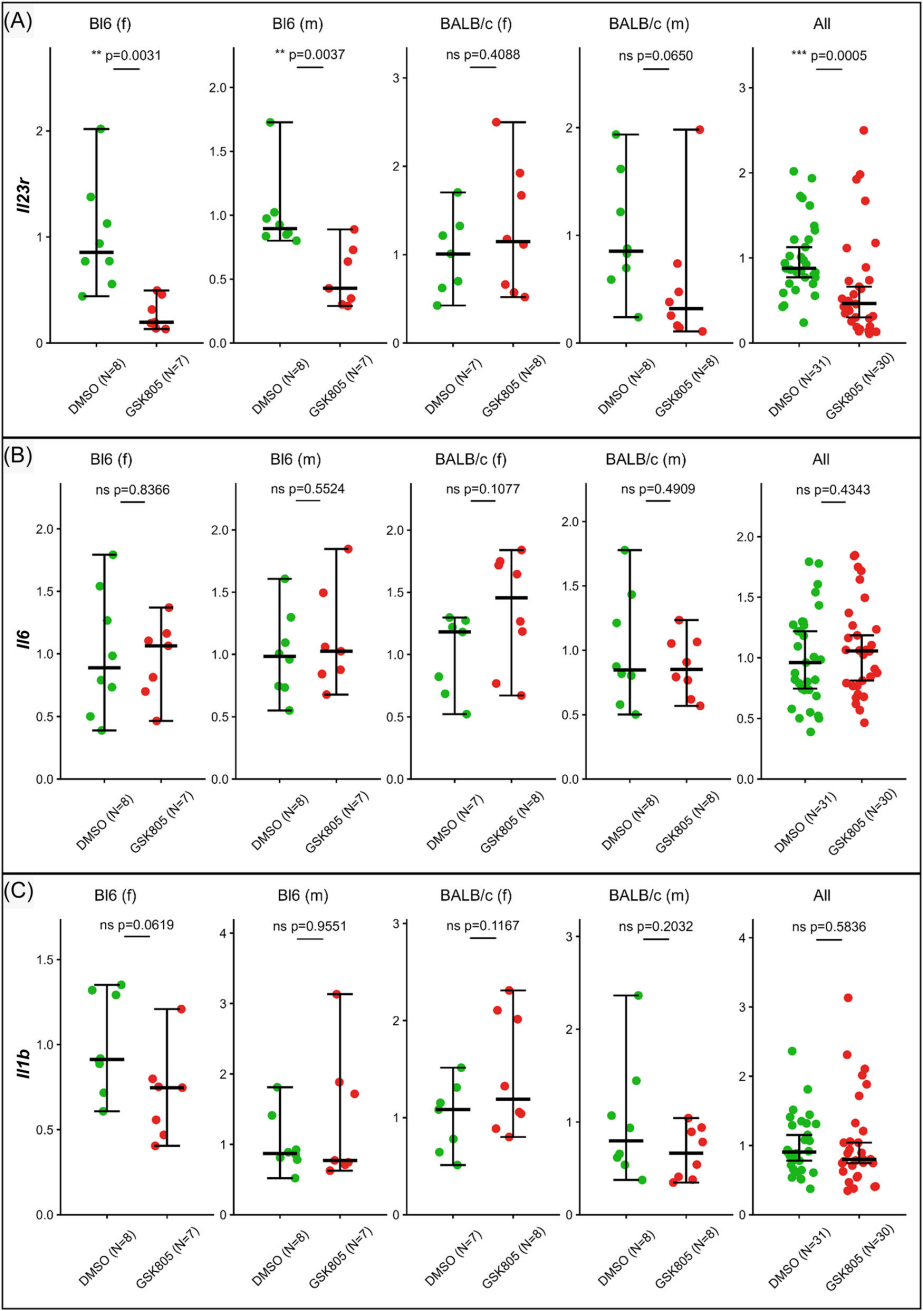
Effects on an interleukin receptor and interleukins. Evaluation of *Il23r* (A), *Il6* (B), and *Il1b* (C) expression across strains and sexes in mice with chronic pancreatitis treated with DMSO or GSK805. All data are presented as fold of mean from DMSO treated mice. Exact *p* values are shown above brackets; significance is denoted by **p < 0.01, ***p < 0.001, and ns (not significant). BL6 = C57BL/6J, f = female, m = male.

We also evaluated the effect of GSK805 treatment on CP (Figures 5, 6, 7, 8, Figures S1 and S3, Table S6). We found a significant reduction in collagen I deposition in BL6 males following treatment with GSK805 (Figure 5). However, no significant changes were observed in BL6 female or BALB/c male and female mice (Figure 6A, Table S6). Thus, GSK805 did not significantly reduce collagen I deposition, with a robustness of 75%. When combining all animal cohorts (Figure 6A) and data from both sexes (Figure S3A), no significant decrease of collagen I was observed. Similarly, GSK805 treatment significantly reduce tubular complex^+^ area only in BL6 males (*p* = 0.0205) (Figure 6B), with a robustness of 75% (Table S6). When combining all animal cohorts (Figure 6B) and data from both sexes (Figure S3B), no significant differences were observed.

In addition, the PW/BW ratio was not significantly changed in GSK805‐treated mice, compared with DMSO‐treated controls (Figure 6C, Table S6). This was observed irrespective of sex or mouse strain, highlighting the robustness of 100% (Table S6). When combining all animal cohorts (Figure 6C) and data from both sexes (Figure S3C), no significant decrease of the PW/BW ratio was noticed.

GSK805 also did not significantly alter *Tnf*, *Ifng*, or *Il10* expression during CP (Figure 7A–C, Table S6), which was observed independent from sex and mouse strains, with a robustness of 100% (Table S6, Figure S3D–F).

When analyzing *Il23r* expression, GSK805 significantly reduced expression in female and male BL6, but not in female and male BALB/c mice (Figure 8A, Table S6). Thus, GSK805 significantly reduced *Il23r* expression in the pancreas, with a robustness of 50%, and was BL6 specific (Figure 8A and Figure S3G). When combining data from all animal cohorts, *Il23r* expression was also significantly reduced (Figure 8A). In contrast, GSK805 did not significantly alter *Il6* or *Il1b* expression during CP (Figure 8B,C, Table S6). This was observed independent of sex and mouse strains, with a robustness of 100% (Table S6, Figure S3H,I).

To assess the numeric stability of the study's findings, the data were categorized into 2 × 2 contingency tables, and UFI and RI were assessed in all mice (Tables 1 and 2). Highly significant differences (*p* < 0.0001) were observed between healthy and CP mice when analyzing collagen I, tubular complex area, PW/BW, and *Il6* and *Il1b* expression in all mice. Additionally, high UFI (i.e., >2) and RI (i.e., >2) values indicated numerical stability across these variables (Table 1). Significant differences were also observed when analyzing *Tnf* (*p* = 0.002) and *Il23r* (*p* = 0.013) expression, whereas *Ifng* and *Il10* did not reach significance (*p* = 0.068 and 0.133, respectively). In the case of *Tnf*, UFI was 2 and RI was 1.8, both values slightly below the cutoff, indicating borderline numerical stability. For *Ifng*, *Il10*, and *Il23r*, a UFI of 1 and lower RI (1.22, 1.55, and 1.26, respectively) suggested a lower numerical stability of these data, warranting cautious interpretation. For example, in the case of *Ifng* or *Il10*, although *p* values were 0.068 and 0.1325, respectively, a single misclassification (moving one sample from healthy to CP group) resulted in a significant *p* value.

**TABLE 1:** Indices to describe the numeric stability of data for the healthy and CP mice.

	Groups	Healthy	CP	*p* value	UFI	RI
Collagen I	*n* ≤ median	10	1	0.0001	3	2.49
	*n* > median	10	30			
TBC	*n* ≤ median	20	3	<0.001	8	5.71
	*n* > median	0	28			
PW/BW	*n* ≤ median	10	31	1.45E−05	4	2.91
	*n* > median	10	0			
*Tnf*	*n* ≤ median	10	3	0.0023	2	1.80
	*n* > median	10	28			
*Ifng*	*n* ≤ median	10	7	0.0680	1	1.22
	*n* > median	10	24			
*Il10*	*n* ≤ median	10	8	0.1325	1	1.55
	*n* > median	10	23			
*Il23r*	*n* ≤ median	10	5	0.0130	1	1.26
	*n* > median	10	26			
*Il6*	*n* ≤ median	10	0	1.45E−05	4	2.91
	*n* > median	10	31			
*Il1b*	*n* ≤ median	10	2	0.0006	3	2.13
	*n* > median	10	29			

*Note*: This table compares the number (*n*) of all healthy mice and all mice with chronic pancreatitis (CP) categorized based on whether they fall below or above the median values observed in healthy mice. Statistical significance between groups is indicated by *p* value. The numeric stability is assessed using unit fragility index (UFI) and robustness index (RI) with higher values representing greater stability and lower values indicating increased numerical fragility.

Abbreviations: PW/BW, pancreas weight to body weight ratio; TBC, tubular complex.

**TABLE 2:** Indices to describe the numeric stability of data for the GSK805‐ and DMSO‐treated CP mice.

	Groups	DMSO	GSK	*p* value	UFI	RI
Collagen I	*n* ≤ median	16	21	0.1919	2	2.12
	*n* > median	15	9			
TBC	*n* ≤ median	16	19	0.440	3	5.03
	*n* > median	15	11			
PW/BW	*n* ≤ median	16	11	0.3056	2	3.21
	*n* > median	15	19			
*Tnf*	*n* ≤ median	16	14	0.7999	4	27.05
	*n* > median	15	16			
*Ifng*	*n* ≤ median	16	12	0.4443	3	5.19
	*n* > median	15	18			
*Il10*	*n* ≤ median	16	16	1.000	4	216.10
	*n* > median	15	14			
*Il23r*	*n* ≤ median	16	23	0.0620	1	1.17
	*n* > median	15	7			
*Il6*	*n* ≤ median	16	13	0.6111	3	9.94
	*n* > median	15	17			
*Il1b*	*n* ≤ median	16	18	0.6089	3	9.60
	*n* > median	15	12			

*Note*: This table compares the number (*n*) of all chronic pancreatitis mice treated with DMSO versus all those treated with GSK805 (GSK), categorized based on whether they fall below or above the median values observed in the DMSO‐treated group. Statistical significance between groups is indicated by *p* value. The numeric stability is assessed using unit fragility index (UFI) and robustness index (RI) with higher values representing greater stability and lower values indicating increased numerical fragility.

When comparing all GSK805‐ and DMSO‐treated CP mice (Table 2), no significant differences were observed after categorization in a 2 × 2 contingency table, with the numerical stability of most data high (UFI >2 and RI >2), supporting the conclusion that GSK805 treatment during CP does not affect collagen I deposition, tubular complex area, PW/BW ratio, or *Tnf*, *Ifng*, *Il10*, *Il6*, and *Il1b* expression. Interestingly, the numerical stability of *Il23r* expression level was low (UFI = 1 and RI = 1.17), suggesting that a single misclassification or slightly increased sample number could have resulted in a significant *p* value.

## 4 Discussion

The presented data demonstrate that the modified CP model using only 42 injections (three daily cerulein injections and three times a week over 30 days) reliably induced CP, and that treatment with 30 mg/kg/day GSK805 failed to alleviate disease. To deepen the interpretation of these data, we employed complementary metrics to assess numerical stability, including RC, UFI, and RI (Tables 1 and 2, Tables S5 and S6).

The straightforward approach of calculating the RC (e.g., Table S5) provided a first estimate about generalizability. Specifically, although PW/BW ratio, collagen I deposition, and tubular complex^+^ area are altered by cerulein‐induced pancreatitis (RC = 100%), the induction of individual cytokines is less robust (RC = 50%–75%) and modulated by strain and sex (Table S5 and Figure S2). This is consistent with the observation in CP patients, where pancreatic atrophy and fibrosis are hallmark features and are present in the vast majority of patients, whereas an increase in specific cytokines is more variable [19, 21, 44]. Thus, analyzing the RC in preclinical research can mirror the clinical situation.

Analyzing stratified subgroups does not only quantify robustness but also offers a basis for identifying strain‐specific responses. For example, *Il10* was significantly increased in both female and male BL6 mice, but not in BALB/c mice, during CP (Figure 3C and Figure S2F). In contrast, *Il6* was significantly elevated in both female and male BALB/c mice. In BL6 mice, the increase in *Il6* expression did not reach statistical significance when analyzed by sex but became significant when data from both sexes were combined (Figure 4B and Figure S2F), suggesting a smaller effect size in this strain. Consistent with this, Cohen's *d* was 0.83 in BL6 mice (combined sexes) and 1.47 in BALB/c mice (combined sexes), indicating a more robust effect in the latter (Table S5).

To the best of our knowledge, no systematic comparison of interleukin expression levels between BALB/c and C57BL/6J mice has been reported in the context of cerulein‐induced CP. However, both strains have been compared in acute pancreatitis models, where it was concluded that cerulein‐induced acute pancreatitis is moderate in BALB/c mice but mild in BL6 mice [45]. This finding contrasts with our results, in which BL6 mice exhibited greater pancreatic atrophy, collagen I deposition, and tubular complex formation compared to BALB/c mice (Figure S2A–C). These opposing strain‐specific outcomes suggest that distinct pathophysiological mechanisms effect the severity of acute and CP. This hypothesis is supported by studies targeting, for example, the STAT3 signaling pathway. Although genetic deletion of *Stat3* exacerbates acute pancreatitis [46], pharmacological inhibition of STAT3 with ruxolitinib ameliorates fibrosis and inflammation in CP [47]. These findings reinforce the notion that the mechanisms driving acute and CP can be fundamentally different.

We showed that CP induces *Il23r* expression significantly in BL6 mice but not in BALB/c mice (Figure 4A, Figure S2G). This suggests that inflammation promotes the IL‐23/Th17 axis more in BL6 than in BALB/c mice. To our knowledge, no other studies have been published describing the RORγt target gene *Il23r* in both mouse strains. However, two studies focused on the RORγt target gene *Il17a* using both mouse strains. Ivanov et al. [30] demonstrated that the overexpression of RORγt induces *Il17* in more CD4^+^ T cells in BL6 mice compared to BALB/c mice. Similarly, Mukhopadhyay et al. [48] demonstrated that after induction of colitis *Il17a* expression is significantly higher in the colon of BL6 mice compared to BALB/c mice. Thus, our results here are consistent with these data and strengthen the hypothesis that the activation of the IL‐23/Th17 axis is stronger in BL6 mice than in BALB/c mice in our CP model. Greater activation of the IL‐23/Th17 axis in BL6 mice might also explain why the inhibition of RORγt, a key transcription factor of this axis, reduces *Il23r* expression mainly in the BL6 mouse strain (Figure 8A and Figure S3G). This divergence reinforces the necessity of selecting appropriate animal models that accurately reflect the immunological context of the study, thereby ensuring the translational relevance and reliability of preclinical findings [30, 48, 49, 50].

In addition, using RI and UFI to analyze our data confirmed the interpretation that our animal model induced CP not only in a robust manner independent of sex and mouse strain, but also that the specific findings we reported are numerically stable. For example, applying the cut‐offs for RI >2 and UFI >2 to our data confirmed that CP induced collagen I, tubular complexes, and *Il6* and *Il1b* expression, while it reduced the PW/BW ratio with high numerical stability (see Table 1). This supports the conclusion that a shortened cerulein induction protocol can recapitulate classical CP features [15, 51]. In contrast, expression of markers such as *Ifng*, *Il10*, and *Il23r*, while statistically significant in our CP animal model, exhibited low UFI values (e.g., 1), highlighting the fragility of the results, as a single data shift could alter their statistical significance. Such a layered approach to data analysis might help to distinguish biologically consistent changes from those that might arise from stochastic variability or sampling artifacts [14].

However, using UFI and RI comes with notable limitations. Both methods rely on 2 × 2 contingency tables, necessitating the transformation of continuous variables into dichotomized outcomes. This simplification can lead to a substantial loss of meaningful variation and reducing statistical power [52]. Furthermore, interpretation of UFI becomes tenuous when two outcomes are tied (either both lowest or highest), as it provides no guidance on how to adjust the contingency table in such scenarios [14]. The RI method has its own limitations, most notably its reliance on the Chi‐square test to calculate *p* value. The Chi‐square test is recommended when sample sizes are large and expected cell frequencies are sufficiently high (generally, *n* > 20 and no cell is expected to have fewer than 5 observations). For smaller samples, Cochran [53] recommends using Fisher's exact test instead. But Fisher's exact test cannot handle decimal values that are generated at each iteration by division or multiplication with a decimal factor. This incompatibility means that RI can be unreliable for some small‐sample studies [5, 53].

We demonstrated that a reduced cerulein injection protocol, that is, 3× per day, instead of 6–8× per day, as standardly used [22, 54], is sufficient to induce hallmark features of CP, including pancreatic atrophy, fibrosis, and inflammation, with medium to large effect sizes (Figures 1, 3, and 4, Table S5). Note that we used metamizole as an analgesic because previous studies demonstrated that this drug does not interfere with clinical and histopathological course of cerulein‐induced pancreatitis [55]. The consistency of our findings across different mouse strains and sexes, and robustness scores reaching 100% for key parameters such as collagen I deposition, tubular complex formation, and PW/BW ratio, underscore the reliability of this milder induction protocol for CP. This less aggressive regimen, consisting of only 42 cerulein injections, enables the study of CP pathophysiology while reducing animal burden and workload for researchers. Most established protocols, in contrast, employ a more prolonged and intensive approach, involving 6 and 7 injections per day, twice weekly for 10 weeks (120–140 injections in total) [22, 23, 54]. Although one study using genetically distinct Swiss albino mice achieved CP within 3 weeks [24], the protocol was more intense than the protocol presented herein, requiring 6 injections per day, 3 days a week (54 injections in total).

Although fewer cerulein injections effectively induced features of CP, treatment with GSK805 had minimal impact on the disease phenotype (Figures 6, 7, 8 and Table S6). Key pathological features, including collagen I deposition, PW/BW, and the expression of cytokine genes such as *Tnf*, *Ifng*, *Il10*, *Il6*, and *Il1b*, did not consistently differ between GSK805‐treated mice and vehicle controls. This might be explained by the dosage of 30 mg/kg/day being too low. However, identical or even lower dosages have been effective in studies of aneurysmal subarachnoid hemorrhage (30 mg/kg/day) [56], autoimmune encephalomyelitis (30 mg/kg/day) [38], and intestinal inflammation (10 mg/kg/day) [36]. In addition, we have previously demonstrated that the same dosing regimen resulted in a median concentration of 19.7 µM GSK805 (95% confidence interval: 12.9–23.5 µM) in the liver [37], which substantially exceeds the 1.5 µM concentration shown to elicit strong effects in cell culture [37]. In addition, we observed that GSK805 significantly reduced the expression of *Il23r* in both male and female BL6 mice (Figure S3G and Table S6). Thus, we conclude that the GSK805 dosage used in our study was pharmacologically sufficient to elicit a biological response in BL6 mice. The strain‐specific effect of *Il23r* inhibition likely points to underlying genetic or epigenetic differences between BL6 and BALB/c mice in the regulation or responsiveness of IL‐23 signaling pathways [57].

Even in BL6 mice, inhibition of *Il23r* expression alone failed to improve other characteristics of CP. This suggested that targeting a single transcription factor (e.g., RORγt, targeted by GSK805) may be insufficient to reverse or mitigate the chronic inflammation and tissue remodeling driven by CP's multifactorial pathology, underscoring the need for broader or combination‐based therapeutic strategies [58, 59].

## 5 Conclusion

We showed that a shortened 3× cerulein administration protocol is sufficient to induce a robust model of CP; we also showed that GSK805 has limited efficacy in the model, suggesting that single‐pathway targeting is inadequate for suppressing CP. The most consistent GSK805 effect observed was a significant reduction in *Il23r* expression in BL6 mice, which was associated with large effect sizes. This result highlights the importance of integrating sex and strain differences into preclinical study design, as biological responses can vary substantially across these variables. To the best of our knowledge, this is the first study in a preclinical animal model to integrate qualitative analyses (e.g., RC) and quantitative metrics (e.g., FI and UFI), alongside conventional *p* value calculations. Incorporating RC, RI, and UFI provides a more nuanced foundation for interpreting disease mechanisms and therapeutic responses in preclinical models.

## Author Contributions

Dietmar Zechner: conceptualization, data interpretation, calculating numerical stability or robustness, writing the original draft. Brigitte Vollmar: conceptualization. Annika Thämlitz: experiments, data management, data analysis, data interpretation. Benjamin Schulz: experiments, data management, data analysis. Lorenzo Pilch: experiments, data management, data analysis. Muhammad Imran Khan: data interpretation, calculating numerical stability or robustness, writing the original draft. All authors critically reviewed the manuscript.

## Funding

This study was supported by Deutsche Forschungsgemeinschaft (DFG), FOR2591, Grant/Award Number: ZE 712/1‐2, ZE 712/1‐3, VO 450/15‐2, and VO 450/15‐3.

## Ethics Statement

All animal experiments were approved by the German local authority, the Landesamt für Landwirtschaft, Lebensmittelsicherheit und Fischerei Mecklenburg‐Vorpommern (approval numbers: AZ 2‐022/19 and 1‐070/20) and were conducted in compliance with ethical guidelines and national law.

## Conflicts of Interest

The authors declare no conflicts of interest.

## Supporting information



Table S1 Example of calculating unit fragility index (UFI) for an observed statistical significance of *p* ≤ 0.05.
Table S2 Example of calculating unit fragility index (UFI) for an observed statistical insignificance of *p* > 0.05.
Table S3 Example of calculating the robustness index (RI) for an observed statistical significance of *p* ≤ 0.05.
Table S4 Example of calculating the robustness index (RI) for an observed statistical significance of *p *> 0.05.
Table S5 Cohen's *d* and robustness of conclusion when comparing healthy mice and mice with chronic pancreatitis.
Table S6 Cohen's *d* and robustness of conclusion when comparing DMSO and GSK805 treated mice in a model of chronic pancreatitis.
Figure S1 Collagen I deposition in pancreas.
Figure S2 Induction of chronic pancreatitis (CP) features in C57BL/6J (BL6) and BALB/c mice.
Figure S3 Limited effects of GSK805 treatment on features of chronic pancreatitis.

## Data Availability

Data were provided to the reviewer and uploaded to figshare.com; https://doi.org/10.6084/m9.figshare.30999889. The software code supporting this study is available under https://github.com/mimranmani/statistical‐fragility.git.
