# Supplementary material for: Enhancing Preclinical Rigor: Evaluating Robustness and Numerical Stability in a Chronic Pancreatitis Mouse Model
Source: Ann N Y Acad Sci. 2026 Jun 9;1560(1):e70295. doi: 10.1111/nyas.70295 (PMC13248879; doi:10.1111/nyas.70295)
Supplement: Supplementary file 1 — Table S1 Example of calculating unit fragility index (UFI) for an observed statistical significance of p ≤ 0.05. Table S2 Example of calculating unit fragility index (UFI) for an observed statistical insignificance of p > 0.05. Table S3 Example of calculating the robustness index (RI) for an observed statistical significance of p ≤ 0.05. Table S4 Example of calculating the robustness index (RI) for an observed statistical significance of p > 0.05. Table S5 Cohen's d and robustness of conclusion when comparing healthy mice and mice with chronic pancreatitis. Table S6 Cohen's d and robustness of conclusion when comparing DMSO and GSK805 treated mice in a model of chronic pancreatitis. Figure S1 Collagen I deposition in pancreas. Figure S2 Induction of chronic pancreatitis (CP) features in C57BL/6J (BL6) and BALB/c mice. Figure S3 Limited effects of GSK805 treatment on features of chronic pancreatitis. [file NYAS-1560-0-s001.docx]

**Supplementary Information**

**Supplementary Tables**

**Table S1.** **Example of calculating unit fragility index (UFI) for an observed statistical significance of p ≤ 0.05.**

| **Sup. 1** | **Group** | **healthy** | **CP** | **totals** | **p-value** | **UFI** |
| --- | --- | --- | --- | --- | --- | --- |
| PW/BW | n ≤ median | 10 | 31 | 41 | 1.45E-05 | 4 |
|  | n > median | 10 | 0 | 10 |  |  |
| **totals** | | 20 | 31 | 51 |  | |
| Iteration: 1 | n ≤ median | 11 | 30 | 41 | 0.0004 | 3 |
|  | n > median | 9 | 1 | 10 |  |  |
| **totals** | | 20 | 31 | 51 |  |  |
| Iteration: 2 | n ≤ median | 12 | 29 | 41 | 0.0085 | 2 |
|  | n > median | 8 | 2 | 10 |  |  |
|  | **totals** | 20 | 31 | 51 |  |  |
| Iteration: 3 | n ≤ median | 13 | 28 | 41 | 0.0357 | 1 |
|  | n > median | 7 | 3 | 10 |  |  |
|  | **totals** | 20 | 31 | 51 |  |  |
| Iteration: 4 | n ≤ median | 14 | 27 | 41 | 0.1627 | 0 |
|  | n > median | 6 | 4 | 10 |  |  |
| **totals** | | 20 | 31 | 51 |  | |

The number (n) of healthy mice and mice with chronic pancreatitis (CP) with a specific pancreas weight-to-body weight ratio (PW/BW) are categorized based on whether they fall below or above the median values observed in healthy mice. UFI is defined as the number of iterations needed to increase the p-value (based on chi-squared test) above 0.05. During each iteration the smallest number in the contingency table is incrementally increased by one unit until the findings become insignificant. Simultaneously other numbers in the contingency table are adjusted by one unit to maintain the fixed marginal totals.

**Table S2. Example of calculating unit fragility index (UFI) for an observed statistical insignificance of p > 0.05.**

| **Sup. 2** |  | **DMSO** | **GSK** | **total** | **p-value** | **UFI** |
| --- | --- | --- | --- | --- | --- | --- |
| *Tnf* | n ≤ median | 16 | 14 | 30 | 0.7997 | 4 |
|  | n > median | 15 | 16 | 31 |  |  |
| **totals** | | 31 | 30 | 61 |  |  |
| Iteration: 1 | n ≤ median | 17 | 13 | 30 | 0.4462 | 3 |
|  | n > median | 14 | 17 | 31 |  |  |
|  | | 31 | 30 | 61 |  |  |
| Iteration: 2 | n ≤ median | 18 | 12 | 30 | 0.2035 | 2 |
|  | n > median | 13 | 18 | 31 |  |  |
| **totals** | | 31 | 30 | 61 |  |  |
| Iteration: 3 | n ≤ median | 19 | 11 | 30 | 0.0744 | 1 |
|  | n > median | 12 | 19 | 31 |  |  |
| **totals** | | 31 | 30 | 61 |  |  |
| Iteration: 4 | n ≤ median | 20 | 10 | 30 | 0.0214 | 0 |
|  | n > median | 11 | 20 | 31 |  |  |
| **totals** | | 31 | 30 | 61 |  | |

The number (n) of chronic pancreatitis mice treated with DMSO versus those treated with GSK805 (GSK), are categorized based on whether they fall below or above the median values observed in the DMSO-treated group. UFI is defined as the number of iterations needed to decrease the p-value (based on chi-squared test) below 0.05. During each iteration the largest number in the contingency table is incrementally increased by one unit until the findings become insignificant. Simultaneously other numbers in the contingency table are adjusted by one unit to maintain the fixed marginal totals.

**Table S3. Example of calculating the robustness index (RI) for an observed statistical significance of p ≤ 0.05.**

| *Tnf* (Table 3) |  | healthy | CP | chi-squared test  p-value | RI |
| --- | --- | --- | --- | --- | --- |
|  | n ≤ median | 10 | 3 | 0.0038 | 1.80 |
|  | n > median | 10 | 28 |  |  |
| 1.500 as divisor | n ≤ median | 7 | 2 | 0.0257 |  |
|  | n > median | 7 | 19 |  |  |
| 1.8214 as divisor | n ≤ median | 5 | 2 | 0.0516 |  |
|  | n > median | 5 | 15 |  |  |

The number (n) of healthy mice and mice with chronic pancreatitis (CP) are categorized in a contingency table based on whether they fall below or above the median values observed in healthy mice. RI is the lowest decimal value that changes the p-value (calculated by chi-squared test) to a p>0.05 when all numbers in the contingency table are divided by this divisor (to simplify, the calculated values in the contingency table are rounded to the nearest integer after division).

**Table S4. Example of calculating the robustness index (RI) for an observed statistical significance of p>0.05.**

| *Il10* (table 3) |  | healthy | CP | chi-squared test  p-value | RI |
| --- | --- | --- | --- | --- | --- |
|  | n ≤ median | 10 | 8 | 0.1429 | 1.55 |
|  | n > median | 10 | 23 |  |  |
| 1.5000 as multiplier | n ≤ median | 15 | 12 | 0.0552 |  |
|  | n > median | 15 | 34 |  |  |
| 1.5543 as multiplier | n ≤ median | 16 | 12 | 0.0499 |  |
|  | n > median | 16 | 36 |  |  |

The number (n) of **healthy** mice and mice with chronic pancreatitis (**CP**) are categorized in a contingency table based on whether they fall below or above the median values observed in healthy mice. RI is the lowest decimal value that changes the p-value (calculated by chi-squared test) to p< 0.05 when all numbers in the contingency table are multiplied by this multiplier (to simplify, the calculated values in the contingency table are rounded to the nearest integer).

**Table S5. Cohen’s d and robustness of conclusion when comparing healthy mice and mice with chronic pancreatitis.**

|  | strain | sex | healthy mice (median: 95% CI) | mice with CP (median: 95% CI) | P-value | Cohen's d   (95% CI) | Con- clusion | RC |
| --- | --- | --- | --- | --- | --- | --- | --- | --- |
| Collagen I | Bl6 | f | 0.606: 0.193-2.045 | 6.440: 0.419-17.772 | 0.0367 | 1.35 [0.08, 2.58] | h < CP |  |
|  | Bl6 | m | 0.937: 0.438-1.499 | 43.299: 14.752-75.668 | 0.0025 | 2.23 [0.75, 3.64] | h < CP |  |
|  | BALB/c | f | 0.945: 0.503-1.561 | 7.565: 2.492-18.019 | 0.0049 | 2.11 [0.61, 3.54] | h < CP |  |
|  | BALB/c | m | 1.066: 0.560-1.590 | 4.829: 1.139-10.733 | 0.0128 | 1.69 [0.35, 2.98] | h < CP |  |
|  | Bl6 | All | 0.800: 0.308-1.848 | 16.727: 5.008-48.309 | <0.0001 | 1.29 [0.41, 2.14] | h < CP |  |
|  | BALB/c | All | 1.006: 0.560-1.561 | 7.163: 3.904-9.826 | 0.0002 | 1.84 [0.87, 2.79] | h < CP |  |
|  | **All mice** |  | **0.941: 0.606-1.465** | **8.552: 5.207-16.639** | **<0.0001** | **1.01 [0.41, 1.60]** | **h < CP** | **100** |
| TBC | Bl6 | f | 0.000: 0.000-0.000 | 1.437: 0.210-10.636 | 0.0033 | 1.00 [-0.21, 2.17] | h < CP |  |
|  | Bl6 | m | 0.000: 0.000-0.000 | 1.717: 0.299-24.390 | 0.0033 | 0.72 [-0.45, 1.86] | h < CP |  |
|  | BALB/c | f | 0.000: 0.000-0.000 | 0.654: 0.024-1.586 | 0.0042 | 1.51 [0.16, 2.80] | h < CP |  |
|  | BALB/c | m | 0.000: 0.000-0.000 | 0.021: 0.000-1.441 | 0.0452 | 0.54 [-0.61, 1.66] | h < CP |  |
|  | Bl6 | All | 0.000: 0.000-0.000 | 1.437: 0.470-5.229 | <0.0001 | 0.78 [-0.05, 1.59] | h < CP |  |
|  | BALB/c | All | 0.000: 0.000-0.000 | 0.103: 0.013-1.164 | 0.0004 | 0.97 [0.11, 1.81] | h < CP |  |
|  | **All mice** |  | **0.000: 0.000-0.000** | **0.654: 0.210-1.441** | **<0.0001** | **0.59 [0.01, 1.16]** | **h < CP** | **100** |
| PW/BW | Bl6 | f | 1.007: 0.860-1.089 | 0.454: 0.373-0.536 | <0.0001 | -8.17 [-11.70, -4.61] | h > CP |  |
|  | Bl6 | m | 1.029: 0.809-1.067 | 0.556: 0.464-0.668 | 0.0016 | -5.02 [-7.35, -2.64] | h > CP |  |
|  | BALB/c | f | 0.999: 0.882-1.117 | 0.737: 0.641-0.830 | 0.0002 | -3.44 [-5.28, -1.53] | h > CP |  |
|  | BALB/c | m | 1.028: 0.762-1.194 | 0.785: 0.696-0.907 | 0.0115 | -1.72 [-3.02, -0.37] | h > CP |  |
|  | Bl6 | All | 1.029: 0.860-1.069 | 0.508: 0.449-0.567 | <0.0001 | -5.44 [-7.15, -3.71] | h > CP |  |
|  | BALB/c | All | 1.010: 0.882-1.117 | 0.758: 0.726-0.830 | <0.0001 | -2.34 [-3.37, -1.28] | h > CP |  |
|  | **All mice** |  | **1.024: 0.975-1.066** | **0.643: 0.537-0.737** | **<0.0001** | **-2.58 [-3.33, -1.82]** | **h > CP** | **100** |
| *Tnf* | Bl6 | f | 0.797: 0.367-2.122 | 4.272: 1.959-6.549 | 0.0026 | 2.21 [0.74, 3.62] | h < CP |  |
|  | Bl6 | m | 0.695: 0.561-2.367 | 5.259: 3.224-7.275 | 0.0016 | 3.54 [1.67, 5.35] | h < CP |  |
|  | BALB/c | f | 1.302: 0.260-1.642 | 0.980: 0.624-1.625 | 0.9229 | 0.06 [-1.09, 1.20] | h ≈ CP |  |
|  | BALB/c | m | 0.497: 0.311-2.358 | 3.342: 2.340-7.231 | 0.0042 | 2.05 [0.62, 3.42] | h < CP |  |
|  | Bl6 | All | 0.720: 0.561-2.122 | 4.620: 3.224-6.216 | <0.0001 | 2.76 [1.64, 3.86] | h < CP |  |
|  | BALB/c | All | 0.899: 0.311-1.642 | 2.340: 0.980-3.536 | 0.0217 | 1.01 [0.15, 1.85] | h < CP |  |
|  | **All mice** |  | **0.720: 0.497-1.463** | **3.536: 2.340-4.650** | **<0.0001** | **1.61 [0.96, 2.25]** | **h < CP** | **75** |
| *Ifng* | Bl6 | f | 0.796: 0.542-1.796 | 3.799: 1.639-6.098 | 0.0038 | 2.08 [0.64, 3.46] | h < CP |  |
|  | Bl6 | m | 0.951: 0.316-1.590 | 2.165: 1.414-7.842 | 0.0062 | 1.07 [-0.15, 2.25] | h < CP |  |
|  | BALB/c | f | 1.308: 0.218-1.565 | 0.577: 0.473-1.545 | 0.4318 | -0.51 [-1.66, 0.68] | h ≈ CP |  |
|  | BALB/c | m | 0.720: 0.448-1.849 | 3.147: 0.872-7.550 | 0.0229 | 1.51 [0.20, 2.76] | h < CP |  |
|  | Bl6 | All | 0.935: 0.542-1.590 | 2.694: 1.855-4.515 | <0.0001 | 1.52 [0.61, 2.41] | h < CP |  |
|  | BALB/c | All | 1.014: 0.448-1.565 | 1.545: 0.577-3.306 | 0.1600 | 0.76 [-0.08, 1.58] | h ≈ CP |  |
|  | **All mice** |  | **0.935: 0.584-1.316** | **2.399: 1.545-3.306** | **<0.0001** | **1.12 [0.51, 1.72]** | **h < CP** | **75** |
| *Il10* | Bl6 | f | 1.123: 0.346-1.586 | 3.045: 1.961-4.797 | 0.0003 | 2.93 [1.26, 4.55] | h < CP |  |
|  | Bl6 | m | 0.719: 0.447-1.662 | 3.375: 1.664-17.023 | 0.0016 | 1.08 [-0.15, 2.26] | h < CP |  |
|  | BALB/c | f | 0.996: 0.127-1.958 | 0.354: 0.212-0.721 | 0.0630 | -1.22 [-2.46, 0.06] | h ≈ CP |  |
|  | BALB/c | m | 0.583: 0.120-2.100 | 2.071: 0.496-5.315 | 0.0769 | 1.11 [-0.12, 2.30] | h ≈ CP |  |
|  | Bl6 | All | 0.921: 0.447-1.586 | 3.127: 2.596-4.593 | <0.0001 | 1.13 [0.27, 1.97] | h < CP |  |
|  | BALB/c | All | 0.790: 0.127-1.958 | 0.721: 0.354-2.290 | 0.6047 | 0.43 [-0.38, 1.24] | h ≈ CP |  |
|  | **All mice** |  | **0.857: 0.583-1.467** | **2.596: 1.664-3.549** | **0.0010** | **0.80 [0.21, 1.38]** | **h < CP** | **50** |
| *Il23r* | Bl6 | f | 0.787: 0.374-1.875 | 3.298: 1.701-7.785 | 0.0097 | 1.78 [0.42, 3.09] | h < CP |  |
|  | Bl6 | m | 0.640: 0.348-2.259 | 5.284: 4.733-10.202 | 0.0016 | 3.27 [1.49, 4.99] | h < CP |  |
|  | BALB/c | f | 0.746: 0.196-2.184 | 0.629: 0.265-1.062 | 0.3068 | -0.63 [-1.80, 0.56] | h ≈ CP |  |
|  | BALB/c | m | 0.601: 0.222-2.319 | 2.058: 0.578-4.669 | 0.0611 | 1.19 [-0.05, 2.39] | h ≈ CP |  |
|  | Bl6 | All | 0.760: 0.374-1.875 | 4.975: 2.978-5.760 | <0.0001 | 2.27 [1.24, 3.28] | h < CP |  |
|  | BALB/c | All | 0.673: 0.222-2.184 | 1.062: 0.578-2.115 | 0.2380 | 0.50 [-0.32, 1.30] | h ≈ CP |  |
|  | **All mice** |  | **0.739: 0.557-1.302** | **2.973: 1.678-4.733** | **0.0001** | **1.17 [0.56, 1.78]** | **h < CP** | **50** |
| *Il6* | Bl6 | f | 0.850: 0.382-1.821 | 2.294: 0.764-5.391 | 0.0586 | 1.20 [-0.04, 2.40] | h ≈ CP |  |
|  | Bl6 | m | 0.296: 0.151-3.984 | 1.440: 0.806-2.353 | 0.0932 | 0.43 [-0.71, 1.55] | h ≈ CP |  |
|  | BALB/c | f | 0.862: 0.419-1.961 | 2.158: 0.954-2.369 | 0.0347 | 1.43 [0.10, 2.71] | h < CP |  |
|  | BALB/c | m | 0.819: 0.361-2.081 | 2.550: 1.512-5.356 | 0.0098 | 1.78 [0.41, 3.08] | h < CP |  |
|  | Bl6 | All | 0.526: 0.220-1.821 | 1.554: 1.095-2.378 | 0.0073 | 0.83 [-0.01, 1.64] | h < CP |  |
|  | BALB/c | All | 0.841: 0.419-1.961 | 2.325: 1.512-2.631 | 0.0015 | 1.47 [0.55, 2.36] | h < CP |  |
|  | **All mice** |  | **0.796: 0.382-1.209** | **2.158: 1.502-2.378** | **<0.0001** | **1.10 [0.50, 1.70]** | **h < CP** | **50** |
| *Il1b* | Bl6 | f | 0.666: 0.309-2.623 | 1.909: 1.273-2.828 | 0.0295 | 1.49 [0.19, 2.74] | h < CP |  |
|  | Bl6 | m | 0.662: 0.273-2.648 | 1.598: 0.955-3.325 | 0.1051 | 1.01 [-0.21, 2.18] | h ≈ CP |  |
|  | BALB/c | f | 0.940: 0.367-1.952 | 1.027: 0.485-1.436 | 0.8638 | -0.10 [-1.25, 1.05] | h ≈ CP |  |
|  | BALB/c | m | 0.524: 0.306-2.874 | 1.954: 0.921-5.804 | 0.0451 | 1.02 [-0.19, 2.20] | h < CP |  |
|  | Bl6 | All | 0.664: 0.309-2.623 | 1.776: 1.488-2.705 | 0.0022 | 1.27 [0.40, 2.13] | h < CP |  |
|  | BALB/c | All | 0.649: 0.367-1.952 | 1.320: 0.921-2.298 | 0.0623 | 0.63 [-0.20, 1.44] | h ≈ CP |  |
|  | **All mice** |  | **0.664: 0.514-0.940** | **1.566: 1.320-1.922** | **0.0004** | **0.88 [0.28, 1.46]** | **h < CP** | **50** |

Differences between the groups were characterized by p-value (Student’s t-test or Mann-Whitney U test). TBC data are presented as % of TBC^+^ area, whereas all other data were normalized to the mean of healthy mice with identical sex and genetic background; TBC = tubular complex, PW/BW = pancreas weight to body weight ratio, Bl6 = C57BL/6J, f = female, m = male, CI= confidence interval, h = healthy, CP = DMSO treated mice with chronic pancreatitis, ≈ means H_0_ hypothesis is accepted (no significant difference), RC = Robustness of Conclusion in % across four independent groups, green field = rejected H_0_ hypothesis, red field = accepted H_0_ hypothesis.

**Table S6. Cohen’s d and robustness of conclusion when comparing DMSO and GSK805 treated mice in a model of chronic pancreatitis**

|  | strain | sex | DMSO treated mice (median: 95% CI) | GSK805 treated mice (median: 95% CI) | P-value | Cohen's d   (95% CI) | Con- clusion | RC |
| --- | --- | --- | --- | --- | --- | --- | --- | --- |
| Collagen I | Bl6 | f | 0.731: 0.048-2.017 | 0.695: 0.373-2.573 | 0.9006 | 0.07 [-0.95, 1.08] | D ≈ G |  |
|  | Bl6 | m | 0.999: 0.340-1.745 | 0.423: 0.062-0.952 | 0.0458 | -1.14 [-2.23, -0.02] | D > G |  |
|  | BALB/c | f | 0.870: 0.287-2.073 | 0.739: 0.145-1.455 | 0.2937 | -0.57 [-1.59, 0.48] | D ≈ G |  |
|  | BALB/c | m | 0.848: 0.200-1.884 | 0.670: 0.223-1.727 | 0.5156 | -0.33 [-1.32, 0.66] | D ≈ G |  |
|  | Bl6 | All | 0.889: 0.375-1.745 | 0.654: 0.338-1.030 | 0.3769 | -0.35 [-1.07, 0.37] | D ≈ G |  |
|  | BALB/c | All | 0.870: 0.685-1.200 | 0.685: 0.355-1.053 | 0.2179 | -0.45 [-1.16, 0.27] | D ≈ G |  |
|  | **All mice** |  | **0.883: 0.568-1.200** | **0.670: 0.423-0.871** | **0.0986** | **-0.40 [-0.91, 0.11]** | **D ≈ G** | **75** |
| TBC | Bl6 | f | 0.497: 0.073-3.680 | 0.299: 0.019-4.512 | 1.0000 | 0.09 [-0.93, 1.10] | D ≈ G |  |
|  | Bl6 | m | 0.368: 0.064-5.228 | 0.050: 0.000-0.418 | 0.0205 | -0.68 [-1.71, 0.38] | D > G |  |
|  | BALB/c | f | 0.886: 0.032-2.148 | 0.172: 0.014-3.347 | 0.2319 | -0.44 [-1.46, 0.60] | D ≈ G |  |
|  | BALB/c | m | 0.100: 0.000-6.751 | 1.044: 0.000-8.199 | 0.2630 | 0.32 [-0.67, 1.31] | D ≈ G |  |
|  | Bl6 | All | 0.487: 0.102-1.121 | 0.188: 0.025-1.033 | 0.1201 | -0.27 [-0.99, 0.45] | D ≈ G |  |
|  | BALB/c | All | 0.320: 0.032-1.576 | 0.295: 0.019-1.482 | 0.8897 | 0.10 [-0.61, 0.80] | D ≈ G |  |
|  | **All mice** |  | **0.397: 0.140-0.886** | **0.248: 0.050-0.418** | **0.4190** | **-0.04 [-0.55, 0.46]** | **D ≈ G** | **75** |
| PW/BW | Bl6 | f | 1.013: 0.835-1.197 | 1.066: 0.844-1.261 | 0.4543 | 0.40 [-0.63, 1.42] | D ≈ G |  |
|  | Bl6 | m | 0.968: 0.808-1.163 | 1.076: 0.939-1.216 | 0.2779 | 0.59 [-0.46, 1.61] | D ≈ G |  |
|  | BALB/c | f | 0.985: 0.857-1.109 | 1.047: 0.916-1.141 | 0.2153 | 0.67 [-0.38, 1.71] | D ≈ G |  |
|  | BALB/c | m | 0.978: 0.866-1.129 | 0.924: 0.853-1.095 | 0.3171 | -0.52 [-1.51, 0.49] | D ≈ G |  |
|  | Bl6 | All | 0.995: 0.935-1.096 | 1.069: 0.939-1.216 | 0.1828 | 0.50 [-0.23, 1.22] | D ≈ G |  |
|  | BALB/c | All | 0.985: 0.944-1.104 | 1.035: 0.896-1.093 | 0.9830 | 0.01 [-0.70, 0.71] | D ≈ G |  |
|  | **All mice** |  | **0.987: 0.944-1.074** | **1.040: 0.958-1.076** | **0.3102** | **0.26 [-0.24, 0.77]** | **D ≈ G** | **100** |
| *Tnf* | Bl6 | f | 1.019: 0.467-1.562 | 0.617: 0.412-0.996 | 0.0753 | -1.00 [-2.07, 0.10] | D ≈ G |  |
|  | Bl6 | m | 0.990: 0.607-1.370 | 0.944: 0.708-1.690 | 0.7820 | 0.15 [-0.87, 1.16] | D ≈ G |  |
|  | BALB/c | f | 0.951: 0.606-1.578 | 1.297: 0.789-2.398 | 0.0920 | 0.94 [-0.15, 2.00] | D ≈ G |  |
|  | BALB/c | m | 0.846: 0.592-1.830 | 0.667: 0.566-1.732 | 0.5737 | -0.20 [-1.18, 0.79] | D ≈ G |  |
|  | Bl6 | All | 1.019: 0.746-1.334 | 0.808: 0.532-1.067 | 0.2442 | -0.44 [-1.16, 0.29] | D ≈ G |  |
|  | BALB/c | All | 0.895: 0.627-1.313 | 1.125: 0.647-1.559 | 0.3146 | 0.37 [-0.35, 1.08] | D ≈ G |  |
|  | **All mice** |  | **0.943: 0.761-1.248** | **0.946: 0.686-1.278** | **0.8739** | **0.06 [-0.45, 0.56]** | **D ≈ G** | **100** |
| *Ifng* | Bl6 | f | 0.990: 0.427-1.589 | 0.461: 0.288-1.419 | 0.1963 | -0.70 [-1.74, 0.36] | D ≈ G |  |
|  | Bl6 | m | 0.766: 0.500-2.773 | 1.219: 0.540-2.187 | 0.2810 | 0.23 [-0.79, 1.24] | D ≈ G |  |
|  | BALB/c | f | 0.757: 0.621-2.026 | 1.589: 1.041-4.138 | 0.0721 | 1.15 [0.03, 2.24] | D ≈ G |  |
|  | BALB/c | m | 0.859: 0.238-2.059 | 0.691: 0.357-1.823 | 0.4358 | -0.40 [-1.39, 0.60] | D ≈ G |  |
|  | Bl6 | All | 0.873: 0.550-1.176 | 0.863: 0.407-1.329 | 0.7901 | -0.13 [-0.85, 0.58] | D ≈ G |  |
|  | BALB/c | All | 0.815: 0.626-1.177 | 1.062: 0.649-2.003 | 0.4463 | 0.49 [-0.23, 1.21] | D ≈ G |  |
|  | **All mice** |  | **0.848: 0.656-1.104** | **1.009: 0.733-1.219** | **0.6622** | **0.26 [-0.24, 0.77]** | **D ≈ G** | **100** |
| *Il10* | Bl6 | f | 0.948: 0.610-1.492 | 0.819: 0.370-1.330 | 0.2746 | -0.59 [-1.62, 0.46] | D ≈ G |  |
|  | Bl6 | m | 0.639: 0.315-3.223 | 0.599: 0.391-1.324 | 0.8665 | -0.37 [-1.39, 0.66] | D ≈ G |  |
|  | BALB/c | f | 0.830: 0.498-1.692 | 2.070: 0.558-4.067 | 0.0448 | 1.15 [0.03, 2.24] | D < G |  |
|  | BALB/c | m | 0.792: 0.190-2.033 | 0.595: 0.236-1.950 | 0.4418 | -0.44 [-1.42, 0.56] | D ≈ G |  |
|  | Bl6 | All | 0.895: 0.606-1.193 | 0.692: 0.514-1.038 | 0.3997 | -0.41 [-1.13, 0.32] | D ≈ G |  |
|  | BALB/c | All | 0.830: 0.662-1.574 | 0.909: 0.558-2.600 | 0.5717 | 0.48 [-0.24, 1.20] | D ≈ G |  |
|  | **All mice** |  | **0.870: 0.672-1.126** | **0.781: 0.591-1.037** | **0.7800** | **0.18 [-0.32, 0.69]** | **D ≈ G** | **75** |
| *Il23r* | Bl6 | f | 0.855: 0.441-2.018 | 0.196: 0.132-0.495 | 0.0031 | -1.87 [-3.09, -0.61] | D > G |  |
|  | Bl6 | m | 0.895: 0.801-1.727 | 0.428: 0.290-0.889 | 0.0037 | -1.76 [-2.95, -0.52] | D > G |  |
|  | BALB/c | f | 1.009: 0.425-1.704 | 1.146: 0.520-2.499 | 0.4088 | 0.44 [-0.59, 1.46] | D ≈ G |  |
|  | BALB/c | m | 0.853: 0.240-1.936 | 0.318: 0.108-1.981 | 0.0650 | -0.79 [-1.80, 0.24] | D ≈ G |  |
|  | Bl6 | All | 0.895: 0.772-1.127 | 0.333: 0.187-0.638 | <0.0001 | -1.81 [-2.65, -0.94] | D > G |  |
|  | BALB/c | All | 0.877: 0.623-1.325 | 0.617: 0.255-1.671 | 0.2995 | -0.16 [-0.86, 0.55] | D ≈ G |  |
|  | **All mice** |  | **0.877: 0.772-1.127** | **0.467: 0.302-0.662** | **0.0005** | **-0.63 [-1.14, -0.11]** | **D > G** | **50** |
| *Il6* | Bl6 | f | 0.887: 0.389-1.793 | 1.064: 0.465-1.370 | 0.8366 | -0.11 [-1.12, 0.91] | D ≈ G |  |
|  | Bl6 | m | 0.983: 0.551-1.607 | 1.025: 0.679-1.847 | 0.5524 | 0.32 [-0.71, 1.33] | D ≈ G |  |
|  | BALB/c | f | 1.181: 0.522-1.297 | 1.456: 0.672-1.840 | 0.1077 | 0.89 [-0.19, 1.95] | D ≈ G |  |
|  | BALB/c | m | 0.846: 0.502-1.778 | 0.850: 0.569-1.234 | 0.4909 | -0.35 [-1.34, 0.64] | D ≈ G |  |
|  | Bl6 | All | 0.972: 0.735-1.298 | 1.042: 0.701-1.370 | 0.8013 | 0.09 [-0.63, 0.81] | D ≈ G |  |
|  | BALB/c | All | 0.873: 0.686-1.273 | 1.058: 0.768-1.646 | 0.4302 | 0.29 [-0.42, 0.99] | D ≈ G |  |
|  | **All mice** |  | **0.960: 0.747-1.219** | **1.056: 0.813-1.185** | **0.4343** | **0.20 [-0.30, 0.70]** | **D ≈ G** | **100** |
| *Il1b* | Bl6 | f | 0.912: 0.608-1.351 | 0.747: 0.405-1.209 | 0.0619 | -1.06 [-2.13, 0.05] | D ≈ G |  |
|  | Bl6 | m | 0.871: 0.520-1.811 | 0.772: 0.626-3.133 | 0.9551 | 0.53 [-0.52, 1.55] | D ≈ G |  |
|  | BALB/c | f | 1.083: 0.512-1.515 | 1.191: 0.801-2.311 | 0.1167 | 0.87 [-0.21, 1.92] | D ≈ G |  |
|  | BALB/c | m | 0.796: 0.375-2.364 | 0.663: 0.347-1.042 | 0.2032 | -0.67 [-1.67, 0.35] | D ≈ G |  |
|  | Bl6 | All | 0.897: 0.783-1.320 | 0.750: 0.558-1.717 | 0.1794 | 0.07 [-0.65, 0.78] | D ≈ G |  |
|  | BALB/c | All | 0.936: 0.617-1.311 | 0.917: 0.541-1.325 | 0.9845 | 0.10 [-0.61, 0.80] | D ≈ G |  |
|  | **All mice** |  | **0.906: 0.781-1.153** | **0.800: 0.745-1.042** | **0.5836** | **0.08 [-0.42, 0.59]** | **D ≈ G** | **100** |

Differences between the groups were characterized by p-value (Student’s t-test or Mann-Whitney U test). TBC data are presented as % of TBC^+^ area, whereas all other data were normalized to the mean of DMSO treated mice with identical sex and genetic background; TBC = tubular complex, PW/BW = pancreas weight to body weight ratio, Bl6 = C57BL/6J, f = female, m = male, CI= confidence interval, D = mice with chronic pancreatitis treated with DMSO, G = mice with chronic pancreatitis treated with GSK805, ≈ means H_0_ hypothesis is accepted (no significant difference), RC = Robustness of Conclusion in % across four independent groups, green field = rejected H_0_ hypothesis, red field = accepted H_0_ hypothesis.

**Supplementary Figures
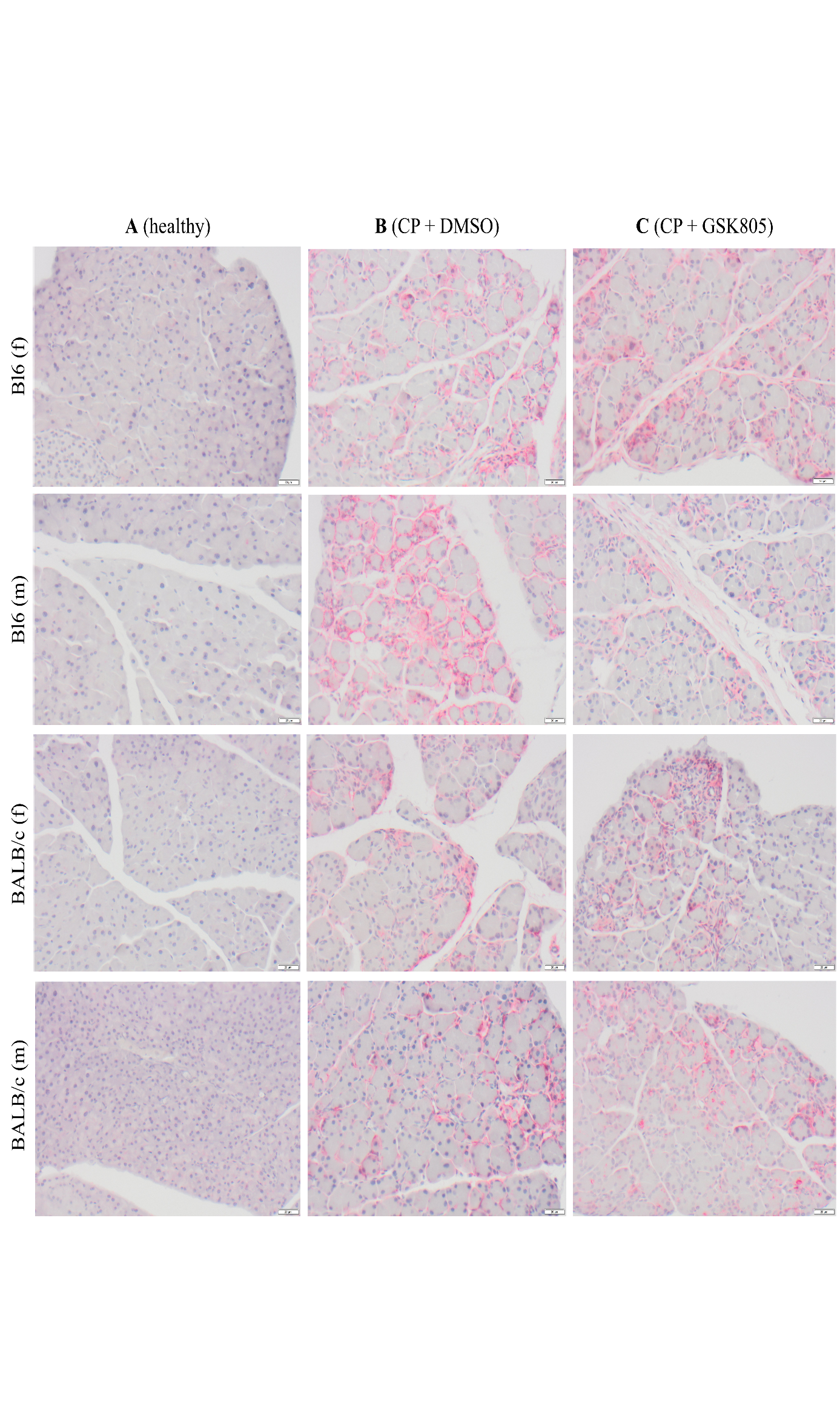
**

**Figure S1**. **Collagen I deposition in pancreas**. Representative pancreatic sections stained for collagen I (red) and counterstained with hematoxylin to visualize nuclei (blue). Columns show tissue from healthy controls (**A**), mice with cerulein-induced chronic pancreatitis (CP) treated with DMSO as vehicle control (**B**), and mice with CP treated with GSK805 (**C**). Bl6 = C57BL/6J, f = female, m = male. Scale bar: 20 µm.

**
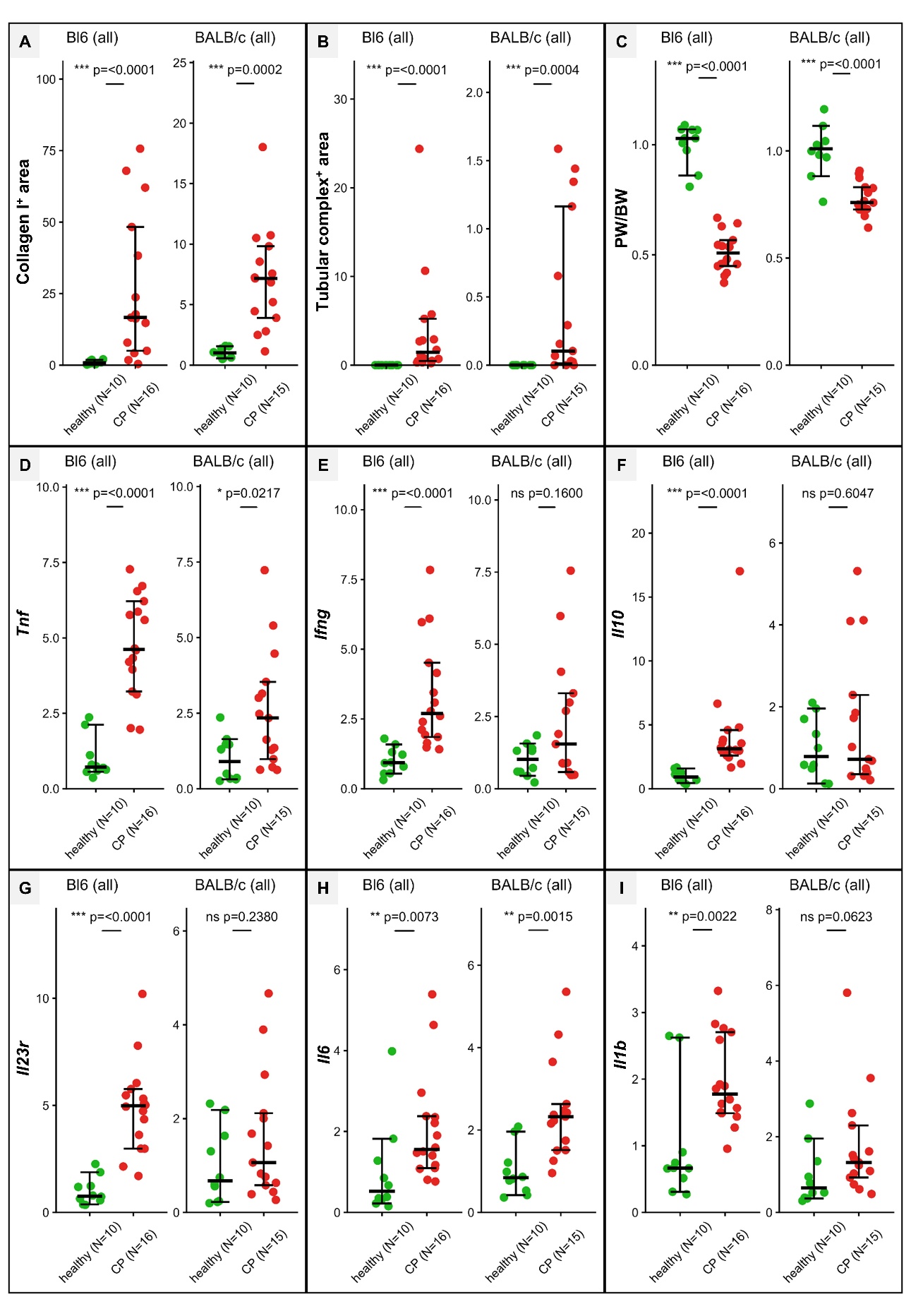
**

**Figure S2**. **Induction of chronic pancreatitis features in C57BL/6J (Bl6) & BALB/c mice.** Collagen I deposition (**A**), tubular complex positive area (**B**), pancreas weight-to-body weight (PW/BW) ratio (**C**), *Tnf* (**D**), *Ifng* (**E**), *Il10* (**F**), *Il23r* (**G**), *Il6* (**H**), and *Il1b* (**I**) expression levels in the pancreas of healthy control mice and mice during chronic pancreatitis (CP). Tubular complex data are presented as % of TBC^+^ area, whereas all other data were normalized to the mean of healthy mice with identical sex and genetic background. Exact p values are shown above brackets; significance is denoted by p<0.05 (*), p<0.01 (**), p<0.001 (***), and ns (not significant).

**
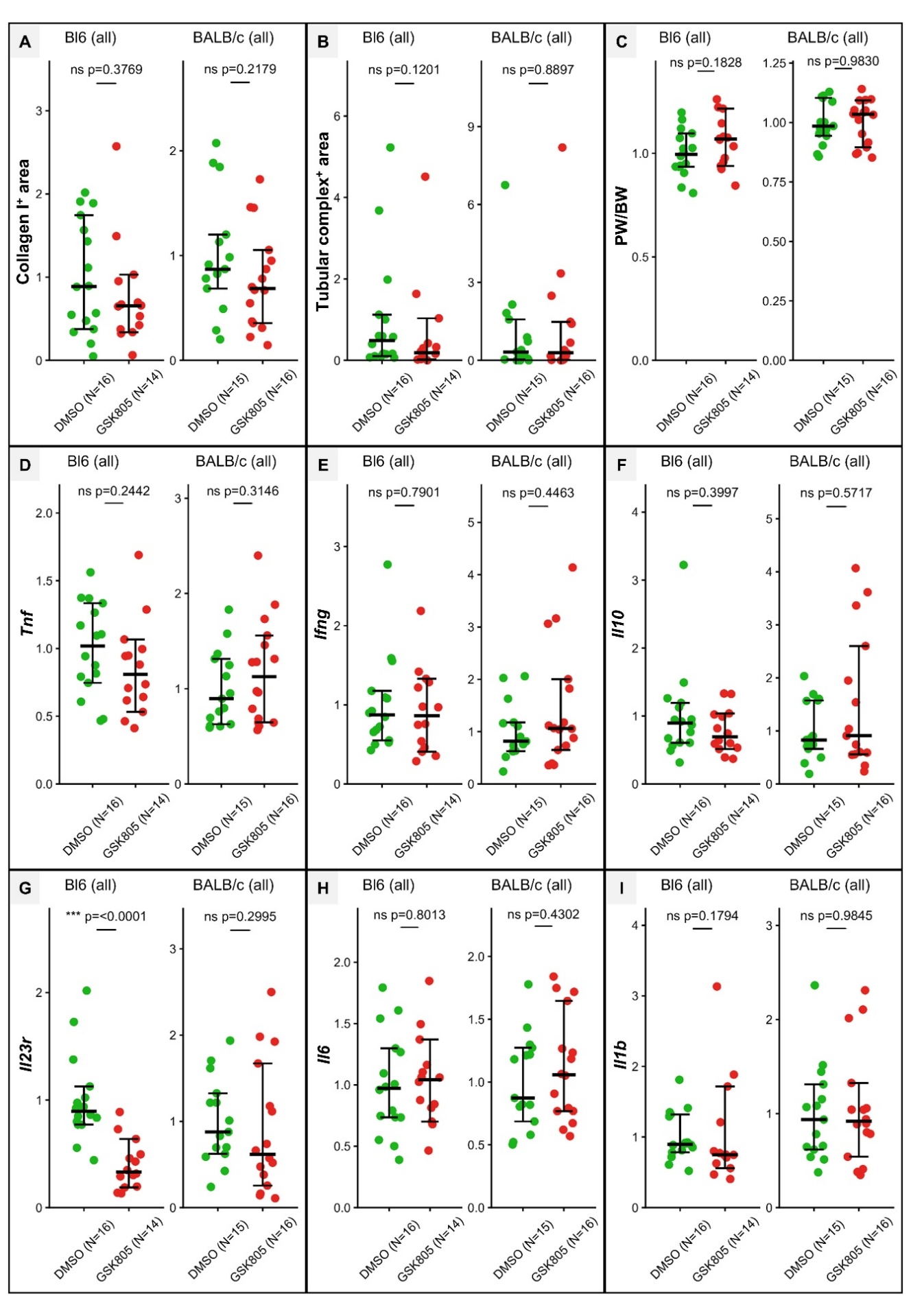
**

**Figure S3**. **Limited effects of GSK805 treatment on features of chronic pancreatitis.** Evaluation of collagen I deposition (**A**), tubular complex positive area (**B**), pancreas weight-to-body weight (PW/BW) ratio (**C**), and expression levels of *Tnf* (**D**), *Ifng* (**E**), *Il10* (**F**), *Il23r* (**G**), *Il6* (**H**), and *Il1b* (**I**), in combined male and female C57BL/6J (Bl6) & BALB/c mice with chronic pancreatitis treated with DMSO (vehicle control) or GSK805. Tubular complex data are presented as % of TBC^+^ area, whereas all other data were normalized to the mean of DMSO treated mice with identical sex and genetic background. Exact p values are shown above brackets; significance is denoted by p<0.05 (*), p<0.01 (**), p<0.001 (***), and ns (not significant).
